# Engineering Concanavalin B to Release Bioactive Peptides against Metabolic Syndrome

**DOI:** 10.3390/foods10071554

**Published:** 2021-07-05

**Authors:** Diego Armando Maldonado-Torres, G. Janet Jara-Romero, Flor de Fátima Rosas-Cárdenas, D. Alejandro Fernández-Velasco, Silvia Luna-Suárez

**Affiliations:** 1 Centro de Investigación en Biotecnología Aplicada, Instituto Politécnico Nacional, CIBA-IPN, Carretera Estatal Tecuexcomac-Tepetitla Km. 1.5, Tepetitla 90700, Mexico; darmanmalto@gmail.com (D.A.M.-T.); janet_16@live.com.mx (G.J.J.-R.); frosasc@ipn.mx (F.d.F.R.-C.); 2 Laboratorio de Fisicoquímica e Ingeniería de Proteínas, Departamento de Bioquímica, Facultad de Medicina, UNAM, Ciudad de México 04510, Mexico

**Keywords:** metabolic syndrome, bioactive peptides, protein engineering, DPPIV, ACEI, antioxidant activity, lipid-lowering

## Abstract

Metabolic syndrome is a severe public health issue characterized by multiple metabolic disturbances. Current treatments prescribe a particular drug for each of them, producing multiple side effects. As a first step towards a more integral approach, we applied our recently described methodology to design single proteins, based in the Concanavalin B scaffold (1CNV), that contain several bioactive peptides (BPs), including antioxidant and lipid-lowering activities as well as inhibitors of dipeptidyl peptidase IV (DPPIV) and the angiotensin converting enzyme. Modified Concanavalin (CNV44), the designed protein that showed the best in silico properties, was expressed in high yields in *E. coli* and purified to homogeneity. After in vitro digestion with gastrointestinal enzymes, all the biological activities tested where higher in CNV44 when compared to the non-modified protein 1CNV, or to other previous reports. The results presented here represent the first in vitro evidence of a modified protein with the potential to treat metabolic syndrome and open the venue for the design of proteins to treat other non-communicable diseases.

## 1. Introduction

Modern lifestyle is responsible for the increase of non-communicable diseases like obesity and its comorbidities [1] such as metabolic syndrome (MS), characterized by multiple metabolic disturbances such as dyslipidemia, insulin resistance, hypertension, pro-thrombotic state, and proinflammatory state, among others. These disorders act synergically to increase the risk of cardiovascular disease (CVD) and stroke. The actual treatment is based on modification of lifestyle, the pharmacological approach usually includes a particular drug for each of the metabolic disturbance, causing many side effects [2].

Functional foods are those with beneficial effects on health beyond nutrition and energetic purposes [3]; the design of functional foods, including proteins and peptides, is a strategy to give options to people with health issues [4].

Bioactive peptides (BPs) have been proposed as candidates to treat MS. BPs are short sequences of amino acids “encrypted” within a parental protein that must be released, by enzymatic hydrolysis or other means, in order to show some positive biological effect [5]. BPs can be released from every food that contains proteins such as milk, meat, fish, cereals, pseudocereals, and legumes. Numerous biological activities from bioactive peptides have been described, with the advantage of not presenting side effects [6]. BPs against all the metabolic disturbances present in MS such as antihypertensive peptides and DPPIV inhibitors have been reported.

Some researchers have designed proteins with the final purpose of carrying bioactive peptides for specific diseases; for example, Takenaka et al. [7] inserted the cholesterol lowering BPs LPYPR in soy pro-glycinin and confirmed that the BP was released after tryptic and chymotryptic digestion. Another approach is to design a completely de novo protein, as reported by Sengqi et al. [8] who built an antihypertensive protein by assembling in tandem, with linker residues between them, previously reported BPs that account for the specificities of gastrointestinal enzymes.

We have previously reported that knowledge of bioactive peptides in combination with protein engineering can be used as a tool with the potential to treat, ameliorate, or prevent MS through the design of a functional compound that enriches a food [9]. These modified proteins can then be used for in vivo trials to test in vivo the biological activity in an organism. As a first step toward this goal, in this work we employed Concanavalin B (CVN) as a scaffold and applied the methodology to the design of a protein, able to carry bioactive peptides, that can be released after an in vitro gastrointestinal digestion with in vitro effects on some metabolic activities related to MS.

## 2. Materials and Methods

### 2.1. Chemicals

All chemicals used were analytical grade. Pepsin from porcine gastric mucosa (>2500 U/mg), pancreatin from porcine pancreas, 3-hydroxy-3-metyl glutaryl-CoA reductase human, NADPH tetrasodium, DL-3Hydroxy-3-methylglutaryl coenzyme A sodium salt hydrate, Angiotensin Converting Enzyme from rabbit lung 2.0 units/mg protein, *N*-Hippuryl-His-Leu hydrate were purchased from Sigma-Aldrich Chemical Company (St. Louis, MO, USA). NdeI and BamHI restriction enzymes were purchased from Thermofisher Scientific, Waltham, MA, USA.

### 2.2. Bioactive Peptides Selection

ACEI peptides and inhibitors of DPPIV peptides were selected from the BIOPEP database [10]; only di- and tripeptides were selected, dipeptides were given priority. These peptides were sorted via their reported IC_50_, and those peptides with in vivo activity were given priority. Antioxidant and lipid-lowering peptides were searched and selected based on the literature. Only those with high activity where selected. To evaluate resistance to gastrointestinal enzymes, selected peptides were submitted to in silico gastrointestinal digestion with BIOPEP and PeptideCutter tools, choosing pepsin pH 1.3, trypsin, and chymotrypsin. Only those that revealed in silico resistance to digestive enzymes were reviewed.

### 2.3. In Silico Design

**Figure 1:**
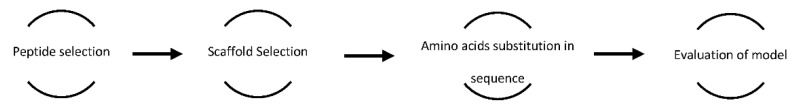
Stages followed to design a protein against metabolic syndrome.

We have previously reported a strategy to design a protein with properties against metabolic syndrome [9]. The Figure 1 summarizes the workflow.

Briefly, we searched in the literature and in the BIOPEP-UWM database for BPs with activity against metabolic disturbances present in metabolic syndrome like angiotensin converting enzyme inhibitory activity (ACE-I), dipeptidyl peptidase IV inhibitory activity (DPPIV-I), antioxidants, and lipid-lowering effects. After obtaining some sequences from vegetable proteins from the UNIPROT database https://www.uniprot.org/ (accessed on 1 February 2017), we searched for the presence of selected BPs into the sequences and calculated the parameter *A* (*A* is the relative frequency of bioactive peptides able to treat metabolic syndrome) with the next equation:

*A* = (a/N) (1)

where “a” is the number of peptides and N is total number of amino acids. The protein with the higher *A* value was selected as scaffold.

To obtain a carrier protein that releases bioactive peptides, we considered the specificities of the following gastrointestinal enzymes, pepsin (Phe or Leu), trypsin (Arg or Lys), and chymotrypsin (aromatics and Leu). In silico evaluation was carried out with Protparam ExPASy-ProtParam tool to assess in vitro stability, VADAR VADAR (wishartlab.com) (accessed on 2–4 March 2021) to evaluate free folding energy (FFE) and Arpeggio Arpeggio (unimelb.edu.au) (accessed on 15–20 March and 10–15 April 2021) to compare changes in molecular interactions.

### 2.4. Expression and Purification

#### 2.4.1. Extraction of Genomic DNA from Canavalia Ensiformis

Genomic DNA was extracted from leaves of *Canavalia ensiformis*. Briefly, a sample of tissue was frozen with liquid nitrogen, mixed with 500 µL of lysis solution (200 mM Tris HCl pH 8.5, 250 mM NaCl, 2.5 mM EDTA and 1% SDS), 500 µL of phenol: chloroform was added and vortexed for one minute and then centrifuged to 12,000 rpm for 10 min, the aqueous phase was separated and mixed with 3M of sodium acetate pH 4.8 (1:10 ratio) and isopropanol (1:2 ratio), the mixture was incubated to −20 °C for 2 h and then centrifuged in the same conditions, supernatant was discarded and pellet was resuspended in 70% of ethanol and centrifuged as previously described, supernatant was discarded, and the pellet resuspended in deionized water.

#### 2.4.2. Primers Design and Extraction of Concanavalin B Gen

With the cDNA sequence reported by Schelesier et al. (1996) [11] we designed the forward 5′ggcatatggacataagttctacagag3′ and reverse 5′ggggatccctaagataacgtataatg3′ primers, with sites for *Nde*I and *Bam*HI restriction enzymes, to amplify Concanavalin B (The PDB Code for Concanavalin B, 1CNV, is used throughout the text as acronym for the protein.); the primers were synthesized by T4Oligo (México). PCR was conducted at a 94 °C denaturation temperature for 2 min and alignment temperature of 57 °C for 35 cycles, amplicon was visualized on a 1% agarose gel, and to confirm the identity was sequenced by dideoxynucleotide label technique by LANBAMA (IPICYT, México). The amplicon was purified from the gel and cloned in pET15b plasmid; this construct was named pET15CNVR.

#### 2.4.3. Expression and Purification of Recombinant Concanavalin (CNVR) and Modified Concanavalin (CNV44)

The gen for CNV44 was designed and sent for synthesis and cloning in pET15b at GenScript ^®^. This construct was named pET15CNV44.

*E. coli* BL21-CodonPlus(DE3)-RIL strain (Stratagene) was transformed by thermal shock with the plasmids pET15CNVR or pET15CNV44 and the proteins (CNVR or CNV44, respectively) were expressed using potato medium [12] at 37 °C for 9 h at constant agitation, protein expression was induced with lactose 0.5% final concentration once the culture reached 0.3 DO at 600 nm, thereafter, the medium was centrifuged at 10,000 rpm for 20 min, pellets were washed with distilled water and later sonicated in phosphate buffer 20 mM pH 7.5, pellets were washed once again and the soluble fraction extracted with phosphate buffer 20 mM + NaCl 0.2 M, insoluble fraction was extracted with urea 6 M. For CNVR, differential precipitation with ammonium sulfate was required. Finally, CNV44 and CNVR were dialyzed against MilliQ water. Final pellets were stored at −10 °C until further analysis. All samples were subjected to 14% SDS-PAGE [13]. The proteins were visualized with Coomassie Brilliant Blue G-250. Quantitative analysis of the recombinant proteins accumulation was carried out by densitometry using Image Lab 4.0 (Bio-Rad).

### 2.5. Simulated Gastrointestinal Digestion

Simulated gastrointestinal digestion (SGID) was based on the report by Vilacundo et al. (2018) [14]; briefly, purified proteins were diluted in water, to start gastric phase pH was adjusted to 2.0 with HCl, pepsin was added in a 250/1 substrate/enzyme ratio, the mix was incubated for 2 h in constant agitation, following by the intestinal phase, where Na_2_CO_3_ was added until pH 7.0 was reached, then pancreatin was added in a ratio 200/1 S/E and was incubated for 12 h, both gastric and intestinal phases was carried out at 37 °C. To stop reaction, the mix was heated to 95 °C for 5 min.

### 2.6. In Vitro Activities

#### 2.6.1. DPPH

DPPH scavenging assay was conducted according to Ajibola et al. (2011) [15], 20 µL of different concentrations of hydrolyzed and unhydrolyzed CNV44 and CNVR were mixed with ethanolic solution of DPPH 150 µM, the reaction was incubated for 30 min in dark at room temperature, then absorbance was followed at 515 nm, percentage of inhibition (% I) was calculated with the next equation:

%I = 1 − (AbsM/Abs0) × 100(2)

where AbsM is sample absorbance and Abs0 is the negative control absorbance.

#### 2.6.2. ABTS

We adapted the 96-well microplate technique reported by Re et al. (1999) [16], a solution containing 7 mM of ABTS and 2.45 mM of potassium persulfate was reposed at room temperature in dark for 16 h before the assay, this solution was diluted in ethanol until the absorbance at 734 nm was 0.7 UA. Different concentrations of hydrolyzed and unhydrolyzed CNV44 and CNVR were tested, 20 µL of each concentration was mixed with 200 µL of ABTS *, after 6 min of incubation at room temperature absorbance was read at 734 nm. Trolox was used as standard. Equation (2) was used to calculate %I.

#### 2.6.3. Fe^++^ Chelation

Fe^++^ chelation capacity was conducted according to Adjimani and Asare (2015) [17] with some modifications. Briefly, 50 µL of FeSO_4_ and 50 µL of sample were mixed and incubated for 10 min, thereafter 100 µL of FerroZine was added and the solution was incubated for 10 more minutes, absorbance was then read at 562 nm, to calculate percentage of quelation (%Q) we used the next equation:

%Q = 1 − (AbsM/Abs0) × 100(3)

where AbsM is sample absorbance and Abs0 is the negative control absorbance.

#### 2.6.4. Inhibition of Angiotensin Converting Enzyme

To evaluate ACEI activity we used the technique reported by Li et al. (2004) [18], 4 µL of samples at different concentrations were mixed with 10 µL of HHL in borate buffer pH 8.3 and incubated at 37 °C for 5 min, then 2 µL of ACE was added and the solution was incubated at the same temperature for 30 more minutes, reaction was stopped with 20 µL of HCl 1M, in order to detect hippuric acid, 48 µL of quinoline and 16 µL of benzenesulfonyl chloride were added and the solution was incubated for 30 min at 30 °C in dark, then absolute ethanol was added and incubated at the same condition of previous step, absorbance was then followed at 492 nm. Percentage inhibition (%I) was calculated with the next equation:

%I = (B − A/B − C) × 100(4)

where A is sample absorbance, B is control absorbance and C is blank absorbance.

#### 2.6.5. Inhibition of DPPIV

To assess DPPIV inhibitory activity, we adapted the technique reported by Lacroix and Li-Chan (2014) [19]. Briefly, 50 µL of samples with variable concentrations were mixed with Gp-pNA 0.2 M in Tris-HCl 100 mM pH8 and incubated for 10 min at 37 °C, reaction started by adding 50 µL of DPPIV enzyme at 0.005 U/mL at same temperature for 1 h, absorbance was measured at 405 nm. %I was calculated using Equation (4).

#### 2.6.6. Inhibition of HMGR

For HMGR inhibition the methodology of Lin et al. (2014) [20] was adapted as follows: different concentrations of samples were mixed with NADPH 200 µM, HMGCoA 10 µM, and 2 µL of human-HMGR at 0.3 mg/mL, final concentrations and 200 µL final volume in phosphate buffer pH 7.4 with EDTA 200 mM, the reaction was conducted at 37 °C and absorbance at 340 nm was measured every 10 min, the results were expressed in µMol of NADPH consumed during the reaction.

### 2.7. Statistical Analysis

All analyses were performed in triplicate and average values were calculated. Statistical significances were evaluated using one-way analysis of variance (ANOVA) and LSD test in SAS 9.0 software.

## 3. Results

### 3.1. Scaffold Selection and Sequence Modification

**Figure 2:**
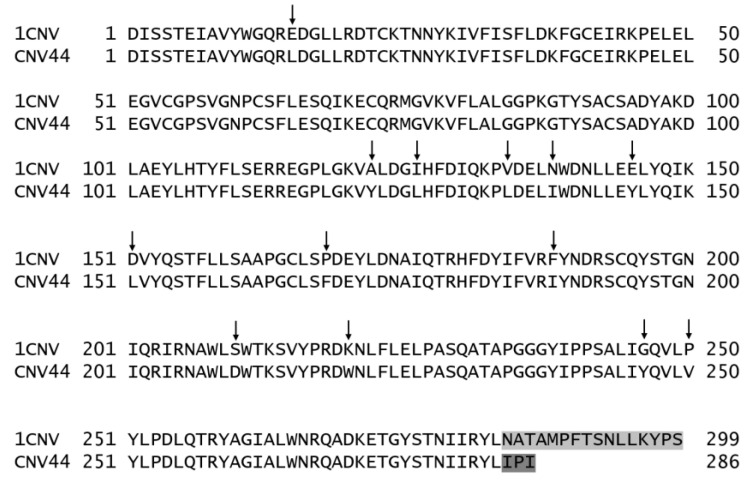
Sequences of Concanavalin B scaffold (1CNV) and Modified Concanavalin (CNV44). Arrows indicate substitution sites. The deleted C-terminal sequence as well as the dipeptidyl peptidase IV (DPPIV) inhibitor that replaces it (IPI) are shown in light gray.

According to our previous peptide selection [9], we searched among some representative vegetable proteins, for those with the major number of BPs in their sequence compared to total number of amino acids (*A* value). Concanavalin B (1CNV) from *Canavalia ensiformis* contain 10 BPs in their sequence (*A* = 0.033) of which seven are antihypertensive (three VY, two EY and two DG), one antioxidant (CG) and two lipid-lowering (DE) (Supplementary Material Table S1) so, we chose Concanavalin B as the scaffold. After in silico gastrointestinal digestion, we observed the profile of peptides with severe reported activities, but none of them were released because they lack the flanking residues required for protease recognition. After identification of BPs, multiple substitutions in adjacent amino acids were made in order to release the BPs on the 1CNV sequence. Ninety-six sequences of CNV with a total of 13 modifications were generated (Supplementary Material Table S2). Because of a lack of secondary structure and BPs the C-terminal, 16 amino acids were deleted and replaced with the tripeptide IPI. Free folding energy and total contacts reveal the major similarity between Concanavalin B and modified concanavalin number 44, from here on named CNV44 (Table 1). CNV44 was evaluated and a new in silico gastrointestinal digestion revealed a new profile of peptides that included all of the selected peptides (Table 2). A comparison between the 1CNV and CNV44 sequences is presented in Figure 2.

### 3.2. Expression and Purification of CNVR and CNV44

#### 3.2.1. CNVR

Genomic DNA from *Canavalia ensiformis* was extracted from fresh leaves. In the agarose gel a 1000 bp fragment was observed (Supplementary Material Figure S1), and sequence analysis of this fragment confirmed its identity as the Concanavalin B gen. The concanavalin B gen was cloned in pET15 plasmid, *E. coli* BL21 CodonPlus (DE3)-RIL strains were further transformed for expression.

**Figure 3:**
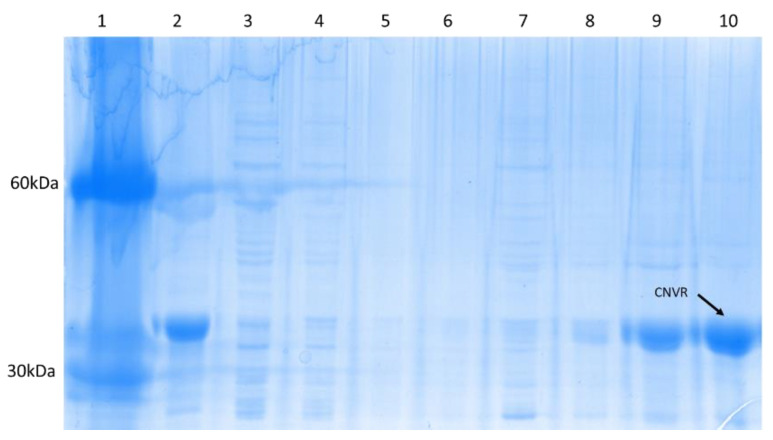
Recombinant Concanavalin (CNVR) Purification. 1 = Molecular marker, 2 = Pellet after expression, 3 = fraction recovered in phosphates buffer, 4–6 = fraction recovered in mercaptoethanol 10 mM, 7–8 = fraction recovered in Urea 1 M, 9–10 = fraction recovered in urea 6 M.

From cells harvested after induction for 6 h, CNVR was extracted in the insoluble fraction, and reached a yield of 1400 ± 10 mg/L, with a productivity of 0.14 mg/h. Recovery and purification are presented in the Figure 3. The purity in urea 6M was 96%.

#### 3.2.2. CNV44

**Figure 4:**
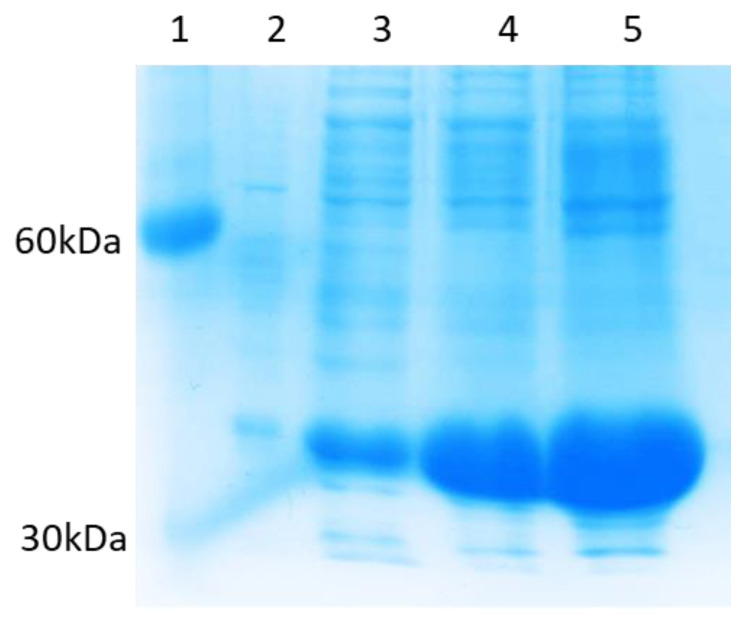
Modified Concanavalin (CNV44) Recovery. 1 = Molecular marker, 2 = Soluble fraction, 3 = Insoluble fraction urea 2 M, 4–5 = Insoluble fraction urea 6 M.

CNV44 was expressed and recovered after 6 h post-induction in insoluble form in urea 6M, the yield was 1460 ± 20 mg/L and productivity of 0.2 mg/h, the purity was about 90% (Figure 4).

**Figure 5:**
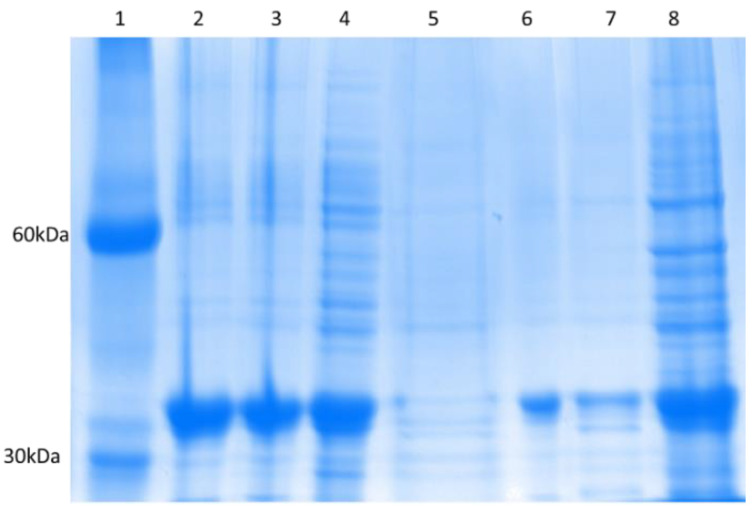
CNV44 Purification. 1 = Molecular marker, 2 = Isoelectric precipitation pH2, 3 = Isoelectric precipitation pH3, 4 = Isoelectric precipitation pH4, 5 = NaCl 1 M, 6 = 4% Ammonium sulfate, 7 = 6% ammonium sulfate, 8 = Starter pellet.

CNV44 was then precipitated by isoelectric precipitation and ammonium sulfate, last one in 4% concentration selectively precipitate CNV44 above of 90% of purity, Figure 5.

### 3.3. In Vitro Activity

#### 3.3.1. Antioxidant Activity

**Figure 6:**
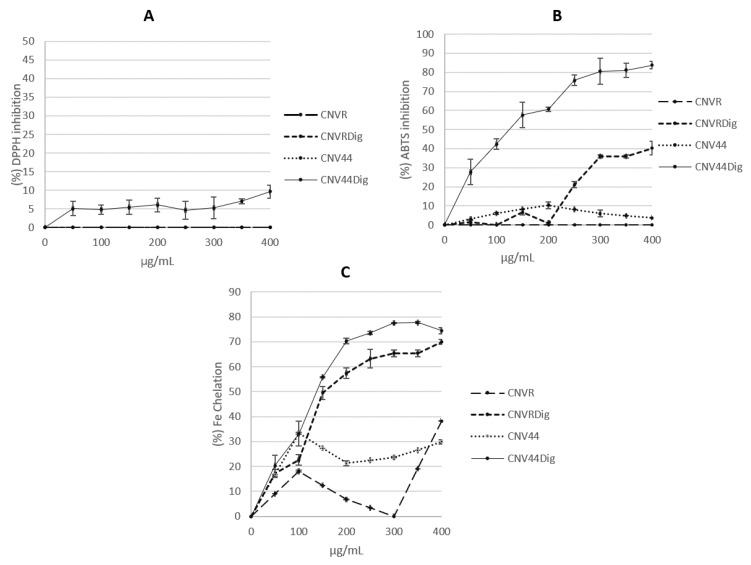
Antioxidant activity of CNVR and CNV44 and their hydrolysates by different techniques, DPPH, ABTS and Iron chelation. (**A**): DPPH radical scavenging, (**B**): ABTS inhibition, (**C**): Iron quelation.

The antioxidant activity assessed with DPPH showed that both CNVR and CNV44 as well as their hydrolysates have a low activity that reaches a maximum of 10% of inhibition at a maximum concentration 400 µg/mL; this maximum activity was found in the hydrolysate of the modified protein (Figure 6).

When testing different concentrations of digested and undigested CNVR and CNV44, ABTS * radical scavenging assay showed greater activity than the DPPH technique. Figure 6 shows that the antioxidant capacity found in digested CNV44 is significantly higher than digested CNV, the maximum inhibition at 400 µg/mL for digested CNV44 is 87.7 ± 2.1% 2-fold higher than digested CNVR, IC50 calculated for digested CNV44 is 131.49 ± 10.44 µg/mL.

For chelating activity, results showed the same tendency observed for ABTS *, low activity was found by undigested CNVR and CNV44, on the other hand, the chelating activity found in hydrolysates reaches 77.5 ± 0.1% and 69.9 ± 1.0% and IC50 values of 131.7 ± 2.4 and 171.09 ± 5.2 for digested CNV44 and digested CNVR, respectively; these differences are statistically different.

#### 3.3.2. ACEI Activity

**Figure 7:**
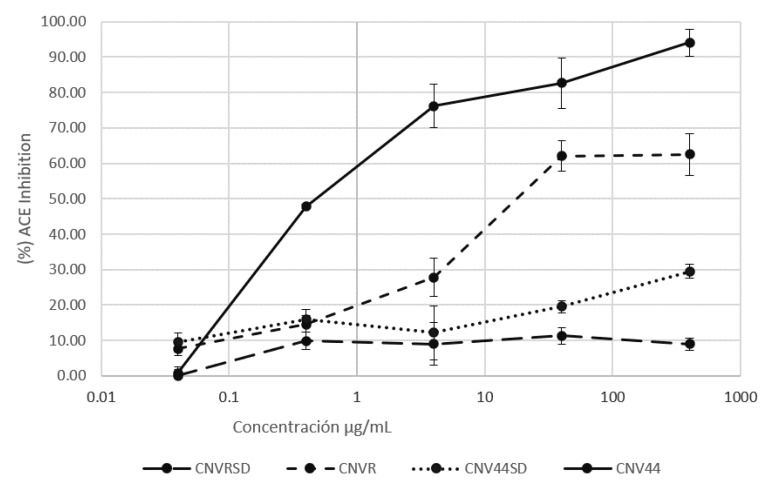
ACEI activity of CNVR and CNV44 and their hydrolysates.

Inhibition of ACE is presented in Figure 7. Unhydrolyzed proteins present low inhibitory activity, CNV44 reached 30% of ACEI at the highest concentration tested (400 µg/mL), higher than CNVR. The hydrolysates of both CNR and CNV44 outperformed their respective non hydrolyzed, digested CNV44 reaches above 94% of ACEI, significantly higher than digested CNVR. IC50 values for both hydrolysates are 4.5 ± 0.6 and 29.3 ± 2.1 for CNV44 and CNVR hydrolysates, respectively, so the modified protein is better than the non-modified one.

#### 3.3.3. DPPIV Inhibition Activity

**Figure 8:**
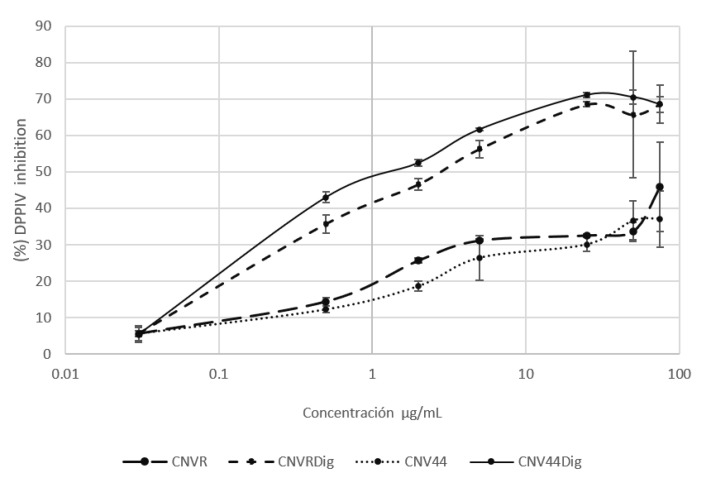
DPPIV inhibitory activity of CNVR and CNV44 and their hydrolysates.

Proteins CNVR, CNV44, and their hydrolysates were tested to measure their capacity to inhibit DPPIV enzyme. The unhydrolyzed protein showed a DPPIV activity 40% lower than its hydrolyzed counterpart, which reaches inhibition percentage above 60% at higher concentrations. IC50 values for hydrolysates are 0.045 ± 0.001 for CNVR hydrolysate and 0.033 ± 0.001 for CNV44 hydrolysate (Figure 8).

#### 3.3.4. HMGCoA Reductase Activity

First, we measured the depletion of NADPH in the test buffer and the results are presented in Supplementary Figure S2. We found that the absorbance at 340 nm remains stable for a period of 30 min, time in which is possible follow the consumption of NADPH utilized for HMGR.

**Figure 9:**
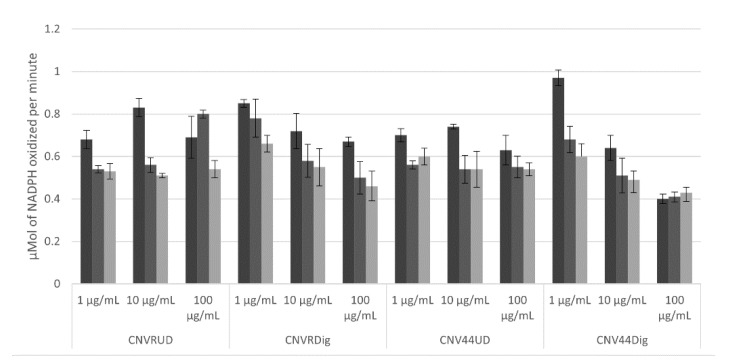
Oxidized NADPH from HMGR in presence of digested and undigested proteins. CNVRUD = Undigested CNVR, CNVRDig = CNVR hydrolyzed, CNV44UD = Undigested CNV44, CNV44Dig = CNV44 hydrolyzed. In dark gray after 10 min, in medium gray after 20 min, and light gray after 30 min.

The consumption of NADPH by HMGR is presented in Figure 9. In general, the lowest consumption is presented in samples at 100 mg/mL, only after 20 min the consumption diminished in most cases. Best results were found in both CNVR and CNV44 hydrolysates in the last one, the inhibition of HMGR is evident in 10 min and stable during all the time lapse.

## 4. Discussion

### 4.1. Scaffold Selection and Sequence Modification

We selected short BPs composed by two or three residues to treat metabolic syndrome because the structure–activity relationship reveals that, in general, while shorter in length their activity is higher than those of longer BPs [23]. On the other hand, protein digestion in intestinal lumen and enterocytes yield 90% of the protein in single amino acids and 10% in di- and tripeptides [24]. Between the BPs selected in this work, IY, DE, and IPI were stated by Iwaniak and Mogut (2020) as potential to treat metabolic syndrome [25], most of the selected BPs are hydrophobic so paracellular absorption via the PEPT1 transporter could be a mechanism for their absorption [26].

Of all the sequences assessed, Concanavalin B showed the higher number of BPs, this protein belongs to the quitinase family with 44% of essential amino acids and a TIM-barrel like structure [27]. To the best of our knowledge Concanavalin B has not been studied in the BPs context.

With the proposed modifications we expect the release of 15 BPs with activity against the major metabolic disturbances present in the metabolic syndrome.

### 4.2. Expression and Purification

The yields and productivities found for CNVR and CNV44 are similar, so modification in the sequence did not alter their expression. Production of proteins with therapeutic capacity for non-communicable disease is a serious concern and expression levels represent a crucial issue to care about. In this work the yield of CNV44 was 1460 mg/L, other food proteins expressed in *E. coli* BL21 strain report expression levels of 480.8 mg/L for the GST-IY-VKY multimer [28] and 675 mg/L for the PTHIKWGD multimer [29]. The expression levels for CNV44 reported in this work (1460 ± 20 mg/L) are superior. In silico work done may have had an impact in this fact, as stability and FFE parameters got good values and, the instability index as a predictor of in vitro environment; values below 40 are considered stable [30]. Most of these proteins have been purified in insoluble form, other strategies to express them in soluble form will be envisioned in future research.

### 4.3. In Vitro Activity

The inhibition of DPPH by the peptides released was really low; however, still, when comparing the proteins, it was observed that digested CNV44 showed the highest inhibition, being significantly different (*p* < 0.05) from the unmodified protein and the two proteins (CNV44 and undigested CNVR).

Regarding ABTS inhibition, the results showed a significant difference (*p* < 0.05) between digested CVN44 (CNV44dig) and the other treatments. There was no significant difference (*p* < 0.05) between undigested CNV44 protein and digested CNVR (CVNRdig). Although at high concentrations (above 200 ug/mL) the digested unmodified protein (CVNRdig) also had favorable results, though much lower (~50%) than the digested modified protein (CNV44dig). The increase in the number of antioxidant peptides may be the reason for the difference found between CNV44 and CNVR, as seen in the peptides presented in the results section. Undigested CNV44 had a very low antioxidant activity, but even so, it presented higher activity than undigested CNVR from 100 ug/mL. CVNR protein did not inhibit free radical production.

Regarding the Fe^+^ chelation, it was observed that digested CNV44 (CNV44Dig) had a significantly (*p* < 0.05) better activity than digested CNVR; from 100 ug/mL, approximately 20% higher Fe^+^ chelation. As with the DPPH and ABTS activities, this could demonstrate the success of the design, as a greater presence of antioxidant peptides would be found, although more studies are necessary to confirm the identity of the released peptides. Between the undigested proteins (CNV44 and CNVR) there was no significant difference.

CNVR and CNV44 hydrolysates increased the antioxidant activity in the three tested techniques. In order to exert their biological activities, bioactive peptides have to be released from their parental proteins sequences because the conformation of the side chains as well as the liberated C- and N-terminal groups responsible for their activity [5], are buried in the structure of the proteins that contain them [31]. The antioxidant activity depends on the techniques used to assess it; while the ABTS and Iron chelation tests showed that hydrolysates have an activity greater than 50%, DPPH does not exceed 10%. It is common, but not a rule, that DPPH and ABTS techniques tend to show similar results, however a couple of studies [32,33] have shown that even though both techniques report on electron transfer [34] higher activities have been obtained with ABTS, as it is observed in this work, especially when lipophilic compounds increase, as it happened with modifications to the CNV sequence where apolar amino acids were introduced.

Research in bioactive peptides is focused on finding the most viable strategies to obtain the highest activities, in general the hydrolysates under different enzymatic hydrolysis schemes are the most used strategies over others, like physical methods or SGID. The maximum percentage of antioxidant activities found in this work at 400 µg/mL were 10% for DPPH, 83.7% for ABTS and 77.5% for chelation. Tang et al. [35] hydrolyzed zein with alcalase and observed 100% inhibition of DPPH and 73.5% of ABTS blenching at 50 mg/mL, the lowest concentration tested was 1 mg/mL where they obtained 12.8% and 64.3% for DPPH and ABTS, respectively. In a recent work, Goncalves et al. (2020) hydrolyzed a cotton seed by-product with alcalase, neutrase, and flavourzyme; they reported activities greater than 95% of DPPH inhibition at concentration of 3 mg/mL [36], that represent 7.5 times the concentration used in this works. ABTS antioxidant activity reported here is similar to the reported by Karamac et al. [37] a result achieved with 2 mg/mL, 5 times higher than those used in present work.

Teixeira et al. [38] reported a carp muscle hydrolysate by SGID and found similar activities than those reported in present work but using a concentration 5 times higher. Likewise, they reported 80% of chelation at 4 mg/mL, a concentration 10 times higher than that used here. Zhang et al. [39] prepared a soy protein hydrolysate with alcalase and after subjected it to SGID, they found increased antioxidant activities except for ABTS. Their results are like those present here; however, their activities were obtained with concentrations between 2.5 to 12.5 times higher than those used in the present work.

Antioxidant peptides have been thoroughly studied. Due to the large number of techniques used to evaluate them, it is very difficult to find trends in terms of their structure-function relationships; nevertheless, one common rule is that smaller peptides exhibit higher activities [40]. It has been proposed that hydrophobic amino acids, specifically aromatics, are key factor for high antioxidant activity [41,42]. In this work, the designed protein was endowed with the DW peptide, in addition to multiple VY, also reported with antioxidant activity [19], both peptides meet the structural requirements previously described.

Regarding the angiotensin converting enzyme inhibitory activity, the statistical analysis showed that CNV44 had an inhibitory effect on ACE, significantly greater (*p* < 0.05) than the recombinant protein without modification (CNVR). A significant difference was found between the undigested proteins with respect to the digested ones; being the higher inhibition in the digested proteins; which agrees with the fact that it is necessary for the peptides to be released from their original sequence, so that they can exert activity. When comparing the two undigested proteins, there was no significant difference between them. This is because the peptides are encrypted in the protein sequences.

Antihypertensive activity is by far the most studied effect of bioactive peptides; all protein sources have been used to generate BPs [43]. In 2002, Pedroche et al. hydrolyzed pea protein isolate with alcalase and flavourzyme, the highest inhibition reported was 40% [44]. In 2009 Je et al. [45] hydrolyzed prickly pear liver with multiple enzymes, their best hydrolysate reached 36% of inhibition. In 2015, Magaña et al. [46] obtained hydrolysates from *Phaseolus lunatus* and some fractions had inhibition percentage above 50% and reported IC50 values in the range of 0.9 to 3.8 µg/mL, Daliri et al. [47] report 88% of ACE inhibition and IC50 of 0.59 mg/mL from soy protein hydrolysates. Garlic protein hydrolysates reported by Gao et al. [48] have IC50 of 0.89 mg/mL; finally, in 2020, Suwannapan et al. [49] studied the effect of SGID on the ACEI activity of rice protein, finding that ACE inhibition does not reach 30%. None of these studies reached the percentage of inhibition or IC50 found for the CNV44 hydrolysate, except for the report by Magaña; however, in the present work, we tested the complete hydrolysates and not fractions as Magaña et al.

According to the design of the CNV44 protein, the presence of ACE inhibitor peptides increased from 5 to 12 including 4 VY, the ACEI peptide with the highest activity reported to date. This increase in the number of ACEI peptides could be responsible for the increase in activity found in the present work.

Regarding the inhibition of the DPPIV enzyme, it was observed that up to 50 ug/mL, there was a significant difference (*p* < 0.05) between digested CNV44 and digested CNVR; although the difference was only ~11%. There was a clear difference between digested and undigested proteins. This is because it is necessary for the peptides to be released from their original sequence, so that they can exert activity. Between undigested CNVR and undigested CNV44 there was no significant difference.

Peptides with DPPIV inhibitory activity have not received much attention, however, some interesting studies are found in the literature; Jia et al. [50] prepared trypsin hydrolysates from α-lactalbumin varying parameters and subsequently subjecting to SGID, they reported IC50 values in the range of 0.41 to 0.61 mg/mL, for samples most extensively hydrolyzed. DPPIV inhibitory activity has been assessed in other hydrolyzed proteins. Hsu et al. [51] reported that skin porcine gelatin hydrolyzed with alcalase reaches an IC50 of 0.06 mg/mL; Lafarga and Hayes [52] reported a bovine lung hydrolyzed with high pressure + alcalase showing an IC50 of 1.43 mg/mL. More recently, two studies [53,54] reported treatments with SGID after enzymatic pretreatments, showing IC50 of 4 mg/mL from sardine muscle and 0.23 mg/mL from boarfish. The hydrolysates of the present work presented an IC50 lower than those reported in literature, only comparable with those obtained by Hsu et al. [51]. The results published by other authors are between 1.4 to 90 times higher than those found for the CNV44 hydrolysate.

The designed protein CNV44, has only one peptide (IPI) exclusive for DPPIV inhibition, the addition of this peptide to the C-term could be responsible for the differences between CNVR and CNV44 hydrolysates, this tripeptide is the peptide with better activity reported against DPPIV, it presents an IC50 of 3.2 µM [55].

HMGR is an enzyme that catalyzes the reduction of HMG-CoA to mevalonate [56]; its inhibition can be detected by NADPH consumption [57]. In the present work, NADPH consumption falls after 10 min and is similar to that reported for Manólio Soares et al. [58]. There was no significant difference in the NADPH oxidation rate between undigested CNVR, digested CNVR, and undigested CNV44. While digested CNV44 showed the lowest oxidation rate of NADPH, being significantly different (*p* < 0.05) than the other proteins, showing higher inhibitory activity of HMGR at 100 ug/mL.

The designed protein has only one lipid-lowering peptide (DE) with previously reported activity, although the impact on this activity can be attributed to this peptide, a dual action by some of the other added peptides cannot be ruled out; furthermore, this peptide is the only one with the structural characteristics and constraints that we established for peptide selection, more research in lipid lowering-peptide is needed in order to improve this particular activity.

It has been reported that soy protein digested with papain + alcalase and subsequent subjected to SGID present antioxidant and ACEI activity as well as DPPIV inhibition [59], showing a maximum ACEI activity of 20.3%, a 2.73 mg/mL IC50 for DPPIV inhibition; and a 5.3 mg/mL IC50 for antioxidant activity. After digestion, all activities increased to values above 70%, always in the order of mg/mL, while in the present work IC50 values are 100 times lower. To the best of our knowledge, the modification of Concanavalin B presented in this work, is the first attempt to engineer a protein to specifically treat or ameliorate metabolic syndrome. Nevertheless, this is only the first step, further studies are required to assess the effect of the modified proteins at the organismal level, because protein hydrolysates assayed in vitro do not necessarily have the same effects in vivo. In addition, all the interpretations of the results described above were related to the properties of the engineered dipeptides, nevertheless the enhanced effects might also be due to other unexpected bioactive sequences released.

## 5. Conclusions

In this work, we present the first in vitro evidence of a modified protein with the potential to treat metabolic syndrome. Modified CNV44 achieved better results than non-modified CNVR and these activities improved after SGID, where CNV44 digested performed better in all the activities tested. Because the biological activities of this protein increase after being digested with gastrointestinal enzymes, this makes it a potential treatment with oral administration; therefore, we developed and tested a new strategy for the design of proteins that can be used for the treatment of other non-communicable disease based on bioactive peptides.

## Figures and Tables

**Table 1:** Comparison between Native Concanavalin B (1CNV) and the 5 modified versions closer to native Concanavalin B (stability, free folding energy, and total contacts). Instability index is better for in vitro conditions at lower numbers (below 40 is considered stable), FFE = Free Folding Energy.

Protein Version	Instability Index	FFE (kcal/mol)	Total Contacts
1CNV	36.38	−287.16	16,138
44	29.97	−294.14	11,519
40	29.71	−293.44	11,510
45	30.4	−293.45	11,521
41	30.14	−292.74	11,508
68	30.4	−292.23	11,467

**Table 2:** Comparison of BPs profiles between 1CNV and CNV44 after in silico gastrointestinal digestion.

Activity	BPs in 1CNV	BPs in CNV44
ACEI	IVF, GK, DY, VR, IR	DG, IVF, VY, GK, IW, IY, IR
DPPIV inhibitors	TY, NW, DN, VR, IR, SW, TK, PY, NR	VY, IW, DN, EY, VR, IR, TK, QV, NR, IPI
Antioxidant	TY, IR, EL	TY, VY ^1^, IR, DW, EL
Lipid lowering	-	DE

^1^ The VY dipeptide has being reported to be antioxidant [21]; however, its main activity is as ACEI [22]. Peptides selected from our previous report are bold [9].

## Data Availability

The data presented in this study are available on request from the corresponding author.

## Supplementary Materials

The following are available online at https://www.mdpi.com/article/10.3390/foods10071554/s1, Table S1: Frequency of selected bioactive peptides in vegetable proteins and their A number. Table S2: The 96 sequences of CNV with a total of 13 modifications generated, Figure S1: PCR product of concanavalin B extracted from leaves, Figure S2: NADPH stability in phosphate buffer pH 7.4 and EDTA 200 mM.
