# Supplementary material for: Engineering Concanavalin B to Release Bioactive Peptides against Metabolic Syndrome
Source: Foods. 2021 Jul 5;10(7):1554. doi: 10.3390/foods10071554 (PMC8307266; doi:10.3390/foods10071554)
Supplement: Supplementary file 1 [file foods-10-01554-s001.zip › foods-1242481-supplementary.pdf]

Table S1. Frequency of selected bioactive peptides in vegetable proteins and their **A** number.

	**Uniprot Entry**	**Sequence Lenght**	**IW**	**VW**	**VY**	**IY**	**EY**	**DG**	**IPI**	**GW**	**CG**	**DW**	**DE**	**Total**	**A**
**Soy**															
**β-conglicinin chain alfa**	P11827	617	0	0	0	1	0	0	0	0	0	0	6	7	0.011
**β-conglicinin chain beta**	P25974	426	0	0	0	1	0	0	0	0	0	0	2	3	0.007
**Albumin 2S**	P19594	137	0	0	0	0	0	0	0	0	0	0	2	2	0.015
**Basic 7S Globulin**	P13917	403	0	1	1	0	1	1	0	0	1	0	0	5	0.012
**Amaranth**															
**11S Globulin**	Q38712	501	0	1	1	2	1	1	0	0	0	0	2	8	0.016
**Oat**															
**Avenin 3**	P80356	201	0	0	1	0	0	0	0	0	0	0	0	1	0.005
**11S Globulin**	Q38780	503	0	0	2	2	1	1	0	0	0	0	2	8	0.016
**Beans**															
**Phaseolin**	P80463	404	0	0	2	1	0	0	0	0	0	0	0	3	0.007
**Globulin-1**	A6YNT0	224	0	0	0	0	0	1	0	0	0	0	2	3	0.013
**Alpha-zein 16**	P04700	242	0	0	1	0	0	0	0	0	0	0	0	1	0.004
**Gamma-zein**	C0P381	267	0	0	0	1	0	0	0	0	2	0	0	3	0.011
**Rice**															
**Prolamin PPROLINE 4E**	Q0DJ45	131	0	1	0	1	0	0	0	0	0	0	0	2	0.015
**Cupincin**	B8AL97	436	0	0	1	0	1	0	0	0	0	0	5	7	0.016
**Globulin**	P29835	164	0	0	0	0	1	0	0	1	0	0	0	2	0.012
**Glutelin**	Q6T725	471	0	0	3	1	2	2	0	0	0	0	3	11	0.023
**Glutelin Type A-2**	P07730	475	0	0	3	1	0	1	0	0	0	0	3	8	0.017
**Glutelin Type B-2**	Q02897	481	0	0	4	1	1	2	0	0	0	0	2	10	0.021
**Maize**															
**Globulin-1 S Allele**	P15590	487	0	0	0	0	0	1	0	0	0	0	2	3	0.006
**22 kDa alpha zein 4**	O48966	245	0	0	0	0	0	0	0	0	0	0	0	0	0.000
**50 kDa gamma zein**	C0P381	267	0	0	0	1	0	0	0	0	2	0	0	3	0.011
**Globulin-2**	Q7M1Z8	431	0	0	1	0	0	0	0	0	0	0	3	4	0.009
**Globulin-1**	A6YNT0	224	0	0	0	0	0	1	0	0	0	0	2	3	0.013

**18 kD delta zein**	Q946V9	190	0	0	0	0	0	0	0	0	0	0	0	0	0.000
**Prolamin PPROL 17**	B6UH22	164	0	0	1	0	0	0	0	0	3	0	0	4	0.024
**Chickpea**															
**Legumin**	Q9SMJ4	475	1	0	0	1	0	1	0	0	0	0	7	10	0.021
**Globulin-1 S Allele**	A0A1S2YZ56	621	0	0	0	1	1	2	0	1	1	0	3	9	0.014
**11S Globulin seed storage**	A0A1S2YGT3	348	1	1	2	0	0	2	0	0	0	0	1	7	0.020
**Glutelin Type-A 2-Like**	A0A1S2YJV5	200	0	1	1	0	0	4	0	0	0	0	0	6	0.030
**Lentil**															
**Albumin S**	P86782	37	0	0	0	0	0	0	0	0	0	0	0	0	0.000
**Jack bean**															
**Concanavalina B**	P46347	299	0	0	3	0	2	2	0	0	1	0	2	10	0.033
**Broad Bean**															
**Legumin type B**	P05190	462	0	0	0	2	2	1	0	0	0	0	1	6	0.013
**Vicilin**	P08438	436	0	0	0	1	2	0	0	0	0	0	2	5	0.011
**Convicilin**	B0BCL8	469	0	0	0	0	1	0	0	0	0	0	4	5	0.011
**Wheat**															
**Glutenin subunit DX5**	P10388	827	0	0	0	0	0	0	0	0	0	0	0	0	0.000
**Avenin-like B1**	Q2A783	267	0	0	0	2	0	0	0	0	0	0	0	2	0.007
**Alpha/Beta Gliadin**	P02863	266	0	0	1	0	0	0	0	0	0	0	0	1	0.004
**Alpha/Beta Gliadin A-I**	P04721	242	0	0	0	0	0	0	0	0	0	0	0	0	0.000
**Alpha/Beta Gliadin A-II**	P04722	271	0	0	1	0	0	0	0	0	0	0	0	1	0.004
**Alpha/Beta Gliadin A-III**	P04723	262	0	0	1	0	0	0	0	0	0	0	0	1	0.004
**Alpha/Beta Gliadin A-IV**	P04724	277	0	0	1	0	0	0	0	0	0	0	0	1	0.004
**Alpha/Beta Gliadin A-V**	P04725	299	0	0	1	0	0	0	0	0	0	0	0	1	0.003
**Glutelin Type A-1**	M7ZVJ6	299	0	1	0	0	0	3	0	0	1	1	0	6	0.020

Parameter **A** is the relative frequency of bioactive peptides that can treat metabolic syndrome.

Table S2. The 96 sequences of CNV with a total of 13 modifications generated.

30. DISSTEIAVYWGQRLDGLLRDTCKTNNYKIVFISFLDKFGCEIRKPELELEGVCGPSVGNPCSFLESQIKECQRMGVKVFLALGGPKGTYSACSADYAKDLAEYLHTYFLSERREGPLGKVYLDGLHFDIQKPLDELIWDNLLEYLYQIKFVYQSTFLLSAAPGCLSFDEYLDNAIQTRHFDYIFVRVYNDRSCQYSTGNIQRIRNAWLIWTKSVYPRDWNLFLELPASQATAPGGGYIPPSALIYQVLVYLPDLQTRYAGIALWNRQADKETGYSTNIIRYLNATAMPFTSNLLKYPS
31. DISSTEIAVYWGQRFDGLLRDTCKTNNYKIVFISFLDKFGCEIRKPELELEGVCGPSVGNPCSFLESQIKECQRMGVKVFLALGGPKGTYSACSADYAKDLAEYLHTYFLSERREGPLGKVYLDGLHFDIQKPLDELIWDNLLEYLYQIKFVYQSTFLLSAAPGCLSFDEYLDNAIQTRHFDYIFVRVYNDRSCQYSTGNIQRIRNAWLIWTKSVYPRDWNLFLELPASQATAPGGGYIPPSALIYQVLVYLPDLQTRYAGIALWNRQADKETGYSTNIIRYLNATAMPFTSNLLKYPS
32. DISSTEIAVYWGQRLDGLLRDTCKTNNYKIVFISFLDKFGCEIRKPELELEGVCGPSVGNPCSFLESQIKECQRMGVKVFLALGGPKGTYSACSADYAKDLAEYLHTYFLSERREGPLGKVYLDGLHFDIQKPLDELIWDNLLEYLYQIKLVYQSTFLLSAAPGCLSFDEYLDNAIQTRHFDYIFVRVYNDRSCQYSTGNIQRIRNAWLIWTKSVYPRDWNLFLELPASQATAPGGGYIPPSALIYQVLVYLPDLQTRYAGIALWNRQADKETGYSTNIIRYLNATAMPFTSNLLKYPS
33. DISSTEIAVYWGQRFDGLLRDTCKTNNYKIVFISFLDKFGCEIRKPELELEGVCGPSVGNPCSFLESQIKECQRMGVKVFLALGGPKGTYSACSADYAKDLAEYLHTYFLSERREGPLGKVYLDGLHFDIQKPLDELIWDNLLEYLYQIKLVYQSTFLLSAAPGCLSFDEYLDNAIQTRHFDYIFVRVYNDRSCQYSTGNIQRIRNAWLIWTKSVYPRDWNLFLELPASQATAPGGGYIPPSALIYQVLVYLPDLQTRYAGIALWNRQADKETGYSTNIIRYLNATAMPFTSNLLKYPS
34. DISSTEIAVYWGQRLDGLLRDTCKTNNYKIVFISFLDKFGCEIRKPELELEGVCGPSVGNPCSFLESQIKECQRMGVKVFLALGGPKGTYSACSADYAKDLAEYLHTYFLSERREGPLGKVYLDGLHFDIQKPLDELIWDNLLEYLYQIKFVYQSTFLLSAAPGCLSFDEYLDNAIQTRHFDYIFVRIYNDRSCQYSTGNIQRIRNAWLIWTKSVYPRDWNLFLELPASQATAPGGGYIPPSALIYQVLVYLPDLQTRYAGIALWNRQADKETGYSTNIIRYLNATAMPFTSNLLKYPS
35. DISSTEIAVYWGQRFDGLLRDTCKTNNYKIVFISFLDKFGCEIRKPELELEGVCGPSVGNPCSFLESQIKECQRMGVKVFLALGGPKGTYSACSADYAKDLAEYLHTYFLSERREGPLGKVYLDGLHFDIQKPLDELIWDNLLEYLYQIKFVYQSTFLLSAAPGCLSFDEYLDNAIQTRHFDYIFVRIYNDRSCQYSTGNIQRIRNAWLIWTKSVYPRDWNLFLELPASQATAPGGGYIPPSALIYQVLVYLPDLQTRYAGIALWNRQADKETGYSTNIIRYLNATAMPFTSNLLKYPS
36. DISSTEIAVYWGQRLDGLLRDTCKTNNYKIVFISFLDKFGCEIRKPELELEGVCGPSVGNPCSFLESQIKECQRMGVKVFLALGGPKGTYSACSADYAKDLAEYLHTYFLSERREGPLGKVYLDGLHFDIQKPLDELIWDNLLEYLYQIKLVYQSTFLLSAAPGCLSFDEYLDNAIQTRHFDYIFVRIYNDRSCQYSTGNIQRIRNAWLIWTKSVYPRDWNLFLELPASQATAPGGGYIPPSALIYQVLVYLPDLQTRYAGIALWNRQADKETGYSTNIIRYLNATAMPFTSNLLKYPS
37. DISSTEIAVYWGQRFDGLLRDTCKTNNYKIVFISFLDKFGCEIRKPELELEGVCGPSVGNPCSFLESQIKECQRMGVKVFLALGGPKGTYSACSADYAKDLAEYLHTYFLSERREGPLGKVYLDGLHFDIQKPLDELIWDNLLEYLYQIKLVYQSTFLLSAAPGCLSFDEYLDNAIQTRHFDYIFVRIYNDRSCQYSTGNIQRIRNAWLIWTKSVYPRDWNLFLELPASQATAPGGGYIPPSALIYQVLVYLPDLQTRYAGIALWNRQADKETGYSTNIIRYLNATAMPFTSNLLKYPS
38. DISSTEIAVYWGQRLDGLLRDTCKTNNYKIVFISFLDKFGCEIRKPELELEGVCGPSVGNPCSFLESQIKECQRMGVKVFLALGGPKGTYSACSADYAKDLAEYLHTYFLSERREGPLGKVYLDGLHFDIQKPLDELIWDNLLEYLYQIKFVYQSTFLLSAAPGCLSFDEYLDNAIQTRHFDYIFVRVYNDRSCQYSTGNIQRIRNAWLDWTKSVYPRDWNLFLELPASQATAPGGGYIPPSALIYQVLVYLPDLQTRYAGIALWNRQADKETGYSTNIIRYLNATAMPFTSNLLKYPS
39. DISSTEIAVYWGQRFDGLLRDTCKTNNYKIVFISFLDKFGCEIRKPELELEGVCGPSVGNPCSFLESQIKECQRMGVKVFLALGGPKGTYSACSADYAKDLAEYLHTYFLSERREGPLGKVYLDGLHFDIQKPLDELIWDNLLEYLYQIKFVYQSTFLLSAAPGCLSFDEYLDNAIQTRHFDYIFVRVYNDRSCQYSTGNIQRIRNAWLDWTKSVYPRDWNLFLELPASQATAPGGGYIPPSALIYQVLVYLPDLQTRYAGIALWNRQADKETGYSTNIIRYLNATAMPFTSNLLKYPS
40. DISSTEIAVYWGQRLDGLLRDTCKTNNYKIVFISFLDKFGCEIRKPELELEGVCGPSVGNPCSFLESQIKECQRMGVKVFLALGGPKGTYSACSADYAKDLAEYLHTYFLSERREGPLGKVYLDGLHFDIQKPLDELIWDNLLEYLYQIKLVYQSTFLLSAAPGCLSFDEYLDNAIQTRHFDYIFVRVYNDRSCQYSTGNIQRIRNAWLDWTKSVYPRDWNLFLELPASQATAPGGGYIPPSALIYQVLVYLPDLQTRYAGIALWNRQADKETGYSTNIIRYLNATAMPFTSNLLKYPS
41. DISSTEIAVYWGQRFDGLLRDTCKTNNYKIVFISFLDKFGCEIRKPELELEGVCGPSVGNPCSFLESQIKECQRMGVKVFLALGGPKGTYSACSADYAKDLAEYLHTYFLSERREGPLGKVYLDGLHFDIQKPLDELIWDNLLEYLYQIKLVYQSTFLLSAAPGCLSFDEYLDNAIQTRHFDYIFVRVYNDRSCQYSTGNIQRIRNAWLDWTKSVYPRDWNLFLELPASQATAPGGGYIPPSALIYQVLVYLPDLQTRYAGIALWNRQADKETGYSTNIIRYLNATAMPFTSNLLKYPS
42. DISSTEIAVYWGQRLDGLLRDTCKTNNYKIVFISFLDKFGCEIRKPELELEGVCGPSVGNPCSFLESQIKECQRMGVKVFLALGGPKGTYSACSADYAKDLAEYLHTYFLSERREGPLGKVYLDGLHFDIQKPLDELIWDNLLEYLYQIKFVYQSTFLLSAAPGCLSFDEYLDNAIQTRHFDYIFVRIYNDRSCQYSTGNIQRIRNAWLDWTKSVYPRDWNLFLELPASQATAPGGGYIPPSALIYQVLVYLPDLQTRYAGIALWNRQADKETGYSTNIIRYLNATAMPFTSNLLKYPS

43. DISSTEIAVYWGQRFDGLLRDTCKTNNYKIVFISFLDKFGCEIRKPELELEGVCGPSVGNPCSFLESQIKECQRMGVKVFLALGGPKGTYSACSADYAKDLAEYLHTYFLSERREGPLGKVYLDGLHFDIQK
PLDELIWDNLLEYLYQIKFVYQSTFLLSAAPGCLSFDEYLDNAIQTRHFDYIFVRIYNDRSCQYSTGNIQRIRNAWLDWTKSVYPRDWNLFLELPASQATAPGGGYIPPSALIYQVLVYLPDLQTRYAGIAL
WNRQADKETGYSTNIIRYLNATAMPFTSNLLKYPS

44. DISSTEIAVYWGQRLDGLLRDTCKTNNYKIVFISFLDKFGCEIRKPELELEGVCGPSVGNPCSFLESQIKECQRMGVKVFLALGGPKGTYSACSADYAKDLAEYLHTYFLSERREGPLGKVYLDGLHFDIQK
PLDELIWDNLLEYLYQIKLVYQSTFLLSAAPGCLSFDEYLDNAIQTRHFDYIFVRIYNDRSCQYSTGNIQRIRNAWLDWTKSVYPRDWNLFLELPASQATAPGGGYIPPSALIYQVLVYLPDLQTRYAGIAL
WNRQADKETGYSTNIIRYLNATAMPFTSNLLKYPS

45. DISSTEIAVYWGQRFDGLLRDTCKTNNYKIVFISFLDKFGCEIRKPELELEGVCGPSVGNPCSFLESQIKECQRMGVKVFLALGGPKGTYSACSADYAKDLAEYLHTYFLSERREGPLGKVYLDGLHFDIQK
PLDELIWDNLLEYLYQIKLVYQSTFLLSAAPGCLSFDEYLDNAIQTRHFDYIFVRIYNDRSCQYSTGNIQRIRNAWLDWTKSVYPRDWNLFLELPASQATAPGGGYIPPSALIYQVLVYLPDLQTRYAGIAL
WNRQADKETGYSTNIIRYLNATAMPFTSNLLKYPS

46. DISSTEIAVYWGQRLDGLLRDTCKTNNYKIVFISFLDKFGCEIRKPELELEGVCGPSVGNPCSFLESQIKECQRMGVKVFLALGGPKGTYSACSADYAKDLAEYLHTYFLSERREGPLGKVYLDGLHFDIQK
PLDELIWDNLLEYLYQIKFVYQSTFLLSAAPGCLSFDEYLDNAIQTRHFDYIFVRVYNDRSCQYSTGNIQRIRNAWLGWTKSVYPRDWNLFLELPASQATAPGGGYIPPSALIYQVLVYLPDLQTRYAGIA
LWNRQADKETGYSTNIIRYLNATAMPFTSNLLKYPS

47. DISSTEIAVYWGQRFDGLLRDTCKTNNYKIVFISFLDKFGCEIRKPELELEGVCGPSVGNPCSFLESQIKECQRMGVKVFLALGGPKGTYSACSADYAKDLAEYLHTYFLSERREGPLGKVYLDGLHFDIQK
PLDELIWDNLLEYLYQIKFVYQSTFLLSAAPGCLSFDEYLDNAIQTRHFDYIFVRVYNDRSCQYSTGNIQRIRNAWLGWTKSVYPRDWNLFLELPASQATAPGGGYIPPSALIYQVLVYLPDLQTRYAGIA
LWNRQADKETGYSTNIIRYLNATAMPFTSNLLKYPS

48. DISSTEIAVYWGQRLDGLLRDTCKTNNYKIVFISFLDKFGCEIRKPELELEGVCGPSVGNPCSFLESQIKECQRMGVKVFLALGGPKGTYSACSADYAKDLAEYLHTYFLSERREGPLGKVYLDGLHFDIQK
PLDELIWDNLLEYLYQIKLVYQSTFLLSAAPGCLSFDEYLDNAIQTRHFDYIFVRVYNDRSCQYSTGNIQRIRNAWLGWTKSVYPRDWNLFLELPASQATAPGGGYIPPSALIYQVLVYLPDLQTRYAGIA
LWNRQADKETGYSTNIIRYLNATAMPFTSNLLKYPS

49. DISSTEIAVYWGQRFDGLLRDTCKTNNYKIVFISFLDKFGCEIRKPELELEGVCGPSVGNPCSFLESQIKECQRMGVKVFLALGGPKGTYSACSADYAKDLAEYLHTYFLSERREGPLGKVYLDGLHFDIQK
PLDELIWDNLLEYLYQIKLVYQSTFLLSAAPGCLSFDEYLDNAIQTRHFDYIFVRVYNDRSCQYSTGNIQRIRNAWLGWTKSVYPRDWNLFLELPASQATAPGGGYIPPSALIYQVLVYLPDLQTRYAGIA
LWNRQADKETGYSTNIIRYLNATAMPFTSNLLKYPS

50. DISSTEIAVYWGQRLDGLLRDTCKTNNYKIVFISFLDKFGCEIRKPELELEGVCGPSVGNPCSFLESQIKECQRMGVKVFLALGGPKGTYSACSADYAKDLAEYLHTYFLSERREGPLGKVYLDGLHFDIQK
PLDELIWDNLLEYLYQIKFVYQSTFLLSAAPGCLSFDEYLDNAIQTRHFDYIFVRIYNDRSCQYSTGNIQRIRNAWLGWTKSVYPRDWNLFLELPASQATAPGGGYIPPSALIYQVLVYLPDLQTRYAGIAL
WNRQADKETGYSTNIIRYLNATAMPFTSNLLKYPS

51. DISSTEIAVYWGQRFDGLLRDTCKTNNYKIVFISFLDKFGCEIRKPELELEGVCGPSVGNPCSFLESQIKECQRMGVKVFLALGGPKGTYSACSADYAKDLAEYLHTYFLSERREGPLGKVYLDGLHFDIQK
PLDELIWDNLLEYLYQIKFVYQSTFLLSAAPGCLSFDEYLDNAIQTRHFDYIFVRIYNDRSCQYSTGNIQRIRNAWLGWTKSVYPRDWNLFLELPASQATAPGGGYIPPSALIYQVLVYLPDLQTRYAGIAL
WNRQADKETGYSTNIIRYLNATAMPFTSNLLKYPS

52. DISSTEIAVYWGQRLDGLLRDTCKTNNYKIVFISFLDKFGCEIRKPELELEGVCGPSVGNPCSFLESQIKECQRMGVKVFLALGGPKGTYSACSADYAKDLAEYLHTYFLSERREGPLGKVYLDGLHFDIQK
PLDELIWDNLLEYLYQIKLVYQSTFLLSAAPGCLSFDEYLDNAIQTRHFDYIFVRIYNDRSCQYSTGNIQRIRNAWLGWTKSVYPRDWNLFLELPASQATAPGGGYIPPSALIYQVLVYLPDLQTRYAGIAL
WNRQADKETGYSTNIIRYLNATAMPFTSNLLKYPS

53. DISSTEIAVYWGQRFDGLLRDTCKTNNYKIVFISFLDKFGCEIRKPELELEGVCGPSVGNPCSFLESQIKECQRMGVKVFLALGGPKGTYSACSADYAKDLAEYLHTYFLSERREGPLGKVYLDGLHFDIQK
PLDELIWDNLLEYLYQIKLVYQSTFLLSAAPGCLSFDEYLDNAIQTRHFDYIFVRIYNDRSCQYSTGNIQRIRNAWLGWTKSVYPRDWNLFLELPASQATAPGGGYIPPSALIYQVLVYLPDLQTRYAGIAL
WNRQADKETGYSTNIIRYLNATAMPFTSNLLKYPS

54. DISSTEIAVYWGQRLDGLLRDTCKTNNYKIVFISFLDKFGCEIRKPELELEGVCGPSVGNPCSFLESQIKECQRMGVKVFLALGGPKGTYSACSADYAKDLAEYLHTYFLSERREGPLGKVYLDGLHFDIQK
PLDELGWDNLLEYLYQIKFVYQSTFLLSAAPGCLSFDEYLDNAIQTRHFDYIFVRVYNDRSCQYSTGNIQRIRNAWLIWTKSVYPRDWNLFLELPASQATAPGGGYIPPSALIYQVLVYLPDLQTRYAGIA
LWNRQADKETGYSTNIIRYLNATAMPFTSNLLKYPS

55. DISSTEIAVYWGQRFDGLLRDTCKTNNYKIVFISFLDKFGCEIRKPELELEGVCGPSVGNPCSFLESQIKECQRMGVKVFLALGGPKGTYSACSADYAKDLAEYLHTYFLSERREGPLGKVYLDGLHFDIQK
PLDELGWDNLLEYLYQIKFVYQSTFLLSAAPGCLSFDEYLDNAIQTRHFDYIFVRVYNDRSCQYSTGNIQRIRNAWLIWTKSVYPRDWNLFLELPASQATAPGGGYIPPSALIYQVLVYLPDLQTRYAGIA
LWNRQADKETGYSTNIIRYLNATAMPFTSNLLKYPS

56. DISSTEIAVYWGQRLDGLLRDTCKTNNYKIVFISFLDKFGCEIRKPELELEGVCGPSVGNPCSFLESQIKECQRMGVKVFLALGGPKGTYSACSADYAKDLAEYLHTYFLSERREGPLGKVYLDGLHFDIQK
PLDELGWDNLLEYLYQIKLVYQSTFLLSAAPGCLSFDEYLDNAIQTRHFDYIFVRVYNDRSCQYSTGNIQRIRNAWLIWTKSVYPRDWNLFLELPASQATAPGGGYIPPSALIYQVLVYLPDLQTRYAGIA
LWNRQADKETGYSTNIIRYLNATAMPFTSNLLKYPS

57. DISSTEIAVYWGQRFDGLLRDTCKTNNYKIVFISFLDKFGCEIRKPELELEGVCGPSVGNPCSFLESQIKECQRMGVKVFLALGGPKGTYSACSADYAKDLAEYLHTYFLSERREGPLGKVYLDGLHFDIQK
PLDELGWDNLLEYLYQIKLVYQSTFLLSAAPGCLSFDEYLDNAIQTRHFDYIFVRVYNDRSCQYSTGNIQRIRNAWLIWTKSVYPRDWNLFLELPASQATAPGGGYIPPSALIYQVLVYLPDLQTRYAGIA
LWNRQADKETGYSTNIIRYLNATAMPFTSNLLKYPS

58. DISSTEIAVYWGQRLDGLLRDTCKTNNYKIVFISFLDKFGCEIRKPELELEGVCGPSVGNPCSFLESQIKECQRMGVKVFLALGGPKGTYSACSADYAKDLAEYLHTYFLSERREGPLGKVYLDGLHFDIQK
PLDELGWDNLLEYLYQIKFVYQSTFLLSAAPGCLSFDEYLDNAIQTRHFDYIFVRIYNDRSCQYSTGNIQRIRNAWLIWTKSVYPRDWNLFLELPASQATAPGGGYIPPSALIYQVLVYLPDLQTRYAGIAL
WNRQADKETGYSTNIIRYLNATAMPFTSNLLKYPS

59. DISSTEIAVYWGQRFDGLLRDTCKTNNYKIVFISFLDKFGCEIRKPELELEGVCGPSVGNPCSFLESQIKECQRMGVKVFLALGGPKGTYSACSADYAKDLAEYLHTYFLSERREGPLGKVYLDGLHFDIQK
PLDELGWDNLLEYLYQIKFVYQSTFLLSAAPGCLSFDEYLDNAIQTRHFDYIFVRIYNDRSCQYSTGNIQRIRNAWLIWTKSVYPRDWNLFLELPASQATAPGGGYIPPSALIYQVLVYLPDLQTRYAGIAL
WNRQADKETGYSTNIIRYLNATAMPFTSNLLKYPS

60. DISSTEIAVYWGQRLDGLLRDTCKTNNYKIVFISFLDKFGCEIRKPELELEGVCGPSVGNPCSFLESQIKECQRMGVKVFLALGGPKGTYSACSADYAKDLAEYLHTYFLSERREGPLGKVYLDGLHFDIQK
PLDELGWDNLLEYLYQIKLVYQSTFLLSAAPGCLSFDEYLDNAIQTRHFDYIFVRIYNDRSCQYSTGNIQRIRNAWLIWTKSVYPRDWNLFLELPASQATAPGGGYIPPSALIYQVLVYLPDLQTRYAGIAL
WNRQADKETGYSTNIIRYLNATAMPFTSNLLKYPS

61. DISSTEIAVYWGQRFDGLLRDTCKTNNYKIVFISFLDKFGCEIRKPELELEGVCGPSVGNPCSFLESQIKECQRMGVKVFLALGGPKGTYSACSADYAKDLAEYLHTYFLSERREGPLGKVYLDGLHFDIQK
PLDELGWDNLLEYLYQIKLVYQSTFLLSAAPGCLSFDEYLDNAIQTRHFDYIFVRIYNDRSCQYSTGNIQRIRNAWLIWTKSVYPRDWNLFLELPASQATAPGGGYIPPSALIYQVLVYLPDLQTRYAGIAL
WNRQADKETGYSTNIIRYLNATAMPFTSNLLKYPS

62. DISSTEIAVYWGQRLDGLLRDTCKTNNYKIVFISFLDKFGCEIRKPELELEGVCGPSVGNPCSFLESQIKECQRMGVKVFLALGGPKGTYSACSADYAKDLAEYLHTYFLSERREGPLGKVYLDGLHFDIQK
PLDELGWDNLLEYLYQIKFVYQSTFLLSAAPGCLSFDEYLDNAIQTRHFDYIFVRVYNDRSCQYSTGNIQRIRNAWLDWTKSVYPRDWNLFLELPASQATAPGGGYIPPSALIYQVLVYLPDLQTRYAGIA
LWNRQADKETGYSTNIIRYLNATAMPFTSNLLKYPS

63. DISSTEIAVYWGQRFDGLLRDTCKTNNYKIVFISFLDKFGCEIRKPELELEGVCGPSVGNPCSFLESQIKECQRMGVKVFLALGGPKGTYSACSADYAKDLAEYLHTYFLSERREGPLGKVYLDGLHFDIQK
PLDELGWDNLLEYLYQIKFVYQSTFLLSAAPGCLSFDEYLDNAIQTRHFDYIFVRVYNDRSCQYSTGNIQRIRNAWLDWTKSVYPRDWNLFLELPASQATAPGGGYIPPSALIYQVLVYLPDLQTRYAGIA
LWNRQADKETGYSTNIIRYLNATAMPFTSNLLKYPS

64. DISSTEIAVYWGQRLDGLLRDTCKTNNYKIVFISFLDKFGCEIRKPELELEGVCGPSVGNPCSFLESQIKECQRMGVKVFLALGGPKGTYSACSADYAKDLAEYLHTYFLSERREGPLGKVYLDGLHFDIQK
PLDELGWDNLLEYLYQIKLVYQSTFLLSAAPGCLSFDEYLDNAIQTRHFDYIFVRVYNDRSCQYSTGNIQRIRNAWLDWTKSVYPRDWNLFLELPASQATAPGGGYIPPSALIYQVLVYLPDLQTRYAGIA
LWNRQADKETGYSTNIIRYLNATAMPFTSNLLKYPS

65. DISSTEIAVYWGQRFDGLLRDTCKTNNYKIVFISFLDKFGCEIRKPELELEGVCGPSVGNPCSFLESQIKECQRMGVKVFLALGGPKGTYSACSADYAKDLAEYLHTYFLSERREGPLGKVYLDGLHFDIQK
PLDELGWDNLLEYLYQIKLVYQSTFLLSAAPGCLSFDEYLDNAIQTRHFDYIFVRVYNDRSCQYSTGNIQRIRNAWLDWTKSVYPRDWNLFLELPASQATAPGGGYIPPSALIYQVLVYLPDLQTRYAGIA
LWNRQADKETGYSTNIIRYLNATAMPFTSNLLKYPS

66. DISSTEIAVYWGQRLDGLLRDTCKTNNYKIVFISFLDKFGCEIRKPELELEGVCGPSVGNPCSFLESQIKECQRMGVKVFLALGGPKGTYSACSADYAKDLAEYLHTYFLSERREGPLGKVYLDGLHFDIQK
PLDELGWDNLLEYLYQIKFVYQSTFLLSAAPGCLSFDEYLDNAIQTRHFDYIFVRIYNDRSCQYSTGNIQRIRNAWLDWTKSVYPRDWNLFLELPASQATAPGGGYIPPSALIYQVLVYLPDLQTRYAGIA
LWNRQADKETGYSTNIIRYLNATAMPFTSNLLKYPS

67. DISSTEIAVYWGQRFDGLLRDTCKTNNYKIVFISFLDKFGCEIRKPELELEGVCGPSVGNPCSFLESQIKECQRMGVKVFLALGGPKGTYSACSADYAKDLAEYLHTYFLSERREGPLGKVYLDGLHFDIQK
PLDELGWDNLLEYLYQIKFVYQSTFLLSAAPGCLSFDEYLDNAIQTRHFDYIFVRIYNDRSCQYSTGNIQRIRNAWLDWTKSVYPRDWNLFLELPASQATAPGGGYIPPSALIYQVLVYLPDLQTRYAGIA
LWNRQADKETGYSTNIIRYLNATAMPFTSNLLKYPS

68. DISSTEIAVYWGQRLDGLLRDTCKTNNYKIVFISFLDKFGCEIRKPELELEGVCGPSVGNPCSFLESQIKECQRMGVKVFLALGGPKGTYSACSADYAKDLAEYLHTYFLSERREGPLGKVYLDGLHFDIQK
PLDELGWDNLLEYLYQIKLVYQSTFLLSAAPGCLSFDEYLDNAIQTRHFDYIFVRIYNDRSCQYSTGNIQRIRNAWLDWTKSVYPRDWNLFLELPASQATAPGGGYIPPSALIYQVLVYLPDLQTRYAGIA
LWNRQADKETGYSTNIIRYLNATAMPFTSNLLKYPS

69. DISSTEIAVYWGQRFDGLLRDTCKTNNYKIVFISFLDKFGCEIRKPELELEGVCGPSVGNPCSFLESQIKECQRMGVKVFLALGGPKGTYSACSADYAKDLAEYLHTYFLSERREGPLGKVYLDGLHFDIQK
PLDELGWDNLLEYLYQIKLVYQSTFLLSAAPGCLSFDEYLDNAIQTRHFDYIFVRIYNDRSCQYSTGNIQRIRNAWLDWTKSVYPRDWNLFLELPASQATAPGGGYIPPSALIYQVLVYLPDLQTRYAGIA
LWNRQADKETGYSTNIIRYLNATAMPFTSNLLKYPS

70. DISSTEIAVYWGQRLDGLLRDTCKTNNYKIVFISFLDKFGCEIRKPELELEGVCGPSVGNPCSFLESQIKECQRMGVKVFLALGGPKGTYSACSADYAKDLAEYLHTYFLSERREGPLGKVYLDGLHFDIQK
PLDELGWDNLLEYLYQIKFVYQSTFLLSAAPGCLSFDEYLDNAIQTRHFDYIFVRVYNDRSCQYSTGNIQRIRNAWLGWTKSVYPRDWNLFLELPASQATAPGGGYIPPSALIYQVLVYLPDLQTRYAGIA
LWNRQADKETGYSTNIIRYLNATAMPFTSNLLKYPS

71. DISSTEIAVYWGQRFDGLLRDTCKTNNYKIVFISFLDKFGCEIRKPELELEGVCGPSVGNPCSFLESQIKECQRMGVKVFLALGGPKGTYSACSADYAKDLAEYLHTYFLSERREGPLGKVYLDGLHFDIQK
PLDELGWDNLLEYLYQIKFVYQSTFLLSAAPGCLSFDEYLDNAIQTRHFDYIFVRVYNDRSCQYSTGNIQRIRNAWLGWTKSVYPRDWNLFLELPASQATAPGGGYIPPSALIYQVLVYLPDLQTRYAGIA
LWNRQADKETGYSTNIIRYLNATAMPFTSNLLKYPS

72. DISSTEIAVYWGQRLDGLLRDTCKTNNYKIVFISFLDKFGCEIRKPELELEGVCGPSVGNPCSFLESQIKECQRMGVKVFLALGGPKGTYSACSADYAKDLAEYLHTYFLSERREGPLGKVYLDGLHFDIQK
PLDELGWDNLLEYLYQIKLVYQSTFLLSAAPGCLSFDEYLDNAIQTRHFDYIFVRVYNDRSCQYSTGNIQRIRNAWLGWTKSVYPRDWNLFLELPASQATAPGGGYIPPSALIYQVLVYLPDLQTRYAGIA
LWNRQADKETGYSTNIIRYLNATAMPFTSNLLKYPS

73. DISSTEIAVYWGQRFDGLLRDTCKTNNYKIVFISFLDKFGCEIRKPELELEGVCGPSVGNPCSFLESQIKECQRMGVKVFLALGGPKGTYSACSADYAKDLAEYLHTYFLSERREGPLGKVYLDGLHFDIQK
PLDELGWDNLLEYLYQIKLVYQSTFLLSAAPGCLSFDEYLDNAIQTRHFDYIFVRVYNDRSCQYSTGNIQRIRNAWLGWTKSVYPRDWNLFLELPASQATAPGGGYIPPSALIYQVLVYLPDLQTRYAGIA
LWNRQADKETGYSTNIIRYLNATAMPFTSNLLKYPS

74. DISSTEIAVYWGQRLDGLLRDTCKTNNYKIVFISFLDKFGCEIRKPELELEGVCGPSVGNPCSFLESQIKECQRMGVKVFLALGGPKGTYSACSADYAKDLAEYLHTYFLSERREGPLGKVYLDGLHFDIQK
PLDELGWDNLLEYLYQIKFVYQSTFLLSAAPGCLSFDEYLDNAIQTRHFDYIFVRIYNDRSCQYSTGNIQRIRNAWLGWTKSVYPRDWNLFLELPASQATAPGGGYIPPSALIYQVLVYLPDLQTRYAGIA
LWNRQADKETGYSTNIIRYLNATAMPFTSNLLKYPS

75. DISSTEIAVYWGQRFDGLLRDTCKTNNYKIVFISFLDKFGCEIRKPELELEGVCGPSVGNPCSFLESQIKECQRMGVKVFLALGGPKGTYSACSADYAKDLAEYLHTYFLSERREGPLGKVYLDGLHFDIQK
PLDELGWDNLLEYLYQIKFVYQSTFLLSAAPGCLSFDEYLDNAIQTRHFDYIFVRIYNDRSCQYSTGNIQRIRNAWLGWTKSVYPRDWNLFLELPASQATAPGGGYIPPSALIYQVLVYLPDLQTRYAGIA
LWNRQADKETGYSTNIIRYLNATAMPFTSNLLKYPS

76. DISSTEIAVYWGQRLDGLLRDTCKTNNYKIVFISFLDKFGCEIRKPELELEGVCGPSVGNPCSFLESQIKECQRMGVKVFLALGGPKGTYSACSADYAKDLAEYLHTYFLSERREGPLGKVYLDGLHFDIQK
PLDELGWDNLLEYLYQIKLVYQSTFLLSAAPGCLSFDEYLDNAIQTRHFDYIFVRIYNDRSCQYSTGNIQRIRNAWLGWTKSVYPRDWNLFLELPASQATAPGGGYIPPSALIYQVLVYLPDLQTRYAGIA
LWNRQADKETGYSTNIIRYLNATAMPFTSNLLKYPS

77. DISSTEIAVYWGQRFDGLLRDTCKTNNYKIVFISFLDKFGCEIRKPELELEGVCGPSVGNPCSFLESQIKECQRMGVKVFLALGGPKGTYSACSADYAKDLAEYLHTYFLSERREGPLGKVYLDGLHFDIQK
PLDELGWDNLLEYLYQIKLVYQSTFLLSAAPGCLSFDEYLDNAIQTRHFDYIFVRIYNDRSCQYSTGNIQRIRNAWLGWTKSVYPRDWNLFLELPASQATAPGGGYIPPSALIYQVLVYLPDLQTRYAGIA
LWNRQADKETGYSTNIIRYLNATAMPFTSNLLKYPS

78. DISSTEIAVYWGQRLDGLLRDTCKTNNYKIVFISFLDKFGCEIRKPELELEGVCGPSVGNPCSFLESQIKECQRMGVKVFLALGGPKGTYSACSADYAKDLAEYLHTYFLSERREGPLGKVYLDGLHFDIQK
PFDELIWDNLLEYLYQIKFVYQSTFLLSAAPGCLSFDEYLDNAIQTRHFDYIFVRVYNDRSCQYSTGNIQRIRNAWLIWTKSVYPRDWNLFLELPASQATAPGGGYIPPSALIYQVLVYLPDLQTRYAGIAL
WNRQADKETGYSTNIIRYLNATAMPFTSNLLKYPS

79. DISSTEIAVYWGQRFDGLLRDTCKTNNYKIVFISFLDKFGCEIRKPELELEGVCGPSVGNPCSFLESQIKECQRMGVKVFLALGGPKGTYSACSADYAKDLAEYLHTYFLSERREGPLGKVYLDGLHFDIQK
PFDELIWDNLLEYLYQIKFVYQSTFLLSAAPGCLSFDEYLDNAIQTRHFDYIFVRVYNDRSCQYSTGNIQRIRNAWLIWTKSVYPRDWNLFLELPASQATAPGGGYIPPSALIYQVLVYLPDLQTRYAGIAL
WNRQADKETGYSTNIIRYLNATAMPFTSNLLKYPS

80. DISSTEIAVYWGQRLDGLLRDTCKTNNYKIVFISFLDKFGCEIRKPELELEGVCGPSVGNPCSFLESQIKECQRMGVKVFLALGGPKGTYSACSADYAKDLAEYLHTYFLSERREGPLGKVYLDGLHFDIQK
PFDELIWDNLLEYLYQIKLVYQSTFLLSAAPGCLSFDEYLDNAIQTRHFDYIFVRVYNDRSCQYSTGNIQRIRNAWLIWTKSVYPRDWNLFLELPASQATAPGGGYIPPSALIYQVLVYLPDLQTRYAGIAL
WNRQADKETGYSTNIIRYLNATAMPFTSNLLKYPS

81. DISSTEIAVYWGQRFDGLLRDTCKTNNYKIVFISFLDKFGCEIRKPELELEGVCGPSVGNPCSFLESQIKECQRMGVKVFLALGGPKGTYSACSADYAKDLAEYLHTYFLSERREGPLGKVYLDGLHFDIQK
PFDELIWDNLLEYLYQIKLVYQSTFLLSAAPGCLSFDEYLDNAIQTRHFDYIFVRVYNDRSCQYSTGNIQRIRNAWLIWTKSVYPRDWNLFLELPASQATAPGGGYIPPSALIYQVLVYLPDLQTRYAGIAL
WNRQADKETGYSTNIIRYLNATAMPFTSNLLKYPS

82. DISSTEIAVYWGQRLDGLLRDTCKTNNYKIVFISFLDKFGCEIRKPELELEGVCGPSVGNPCSFLESQIKECQRMGVKVFLALGGPKGTYSACSADYAKDLAEYLHTYFLSERREGPLGKVYLDGLHFDIQK
PFDELIWDNLLEYLYQIKFVYQSTFLLSAAPGCLSFDEYLDNAIQTRHFDYIFVRIYNDRSCQYSTGNIQRIRNAWLIWTKSVYPRDWNLFLELPASQATAPGGGYIPPSALIYQVLVYLPDLQTRYAGIAL
WNRQADKETGYSTNIIRYLNATAMPFTSNLLKYPS

83. DISSTEIAVYWGQRFDGLLRDTCKTNNYKIVFISFLDKFGCEIRKPELELEGVCGPSVGNPCSFLESQIKECQRMGVKVFLALGGPKGTYSACSADYAKDLAEYLHTYFLSERREGPLGKVYLDGLHFDIQK
PFDELIWDNLLEYLYQIKFVYQSTFLLSAAPGCLSFDEYLDNAIQTRHFDYIFVRIYNDRSCQYSTGNIQRIRNAWLIWTKSVYPRDWNLFLELPASQATAPGGGYIPPSALIYQVLVYLPDLQTRYAGIAL
WNRQADKETGYSTNIIRYLNATAMPFTSNLLKYPS

84. DISSTEIAVYWGQRLDGLLRDTCKTNNYKIVFISFLDKFGCEIRKPELELEGVCGPSVGNPCSFLESQIKECQRMGVKVFLALGGPKGTYSACSADYAKDLAEYLHTYFLSERREGPLGKVYLDGLHFDIQK
PFDELIWDNLLEYLYQIKLVYQSTFLLSAAPGCLSFDEYLDNAIQTRHFDYIFVRIYNDRSCQYSTGNIQRIRNAWLIWTKSVYPRDWNLFLELPASQATAPGGGYIPPSALIYQVLVYLPDLQTRYAGIAL
WNRQADKETGYSTNIIRYLNATAMPFTSNLLKYPS

85. DISSTEIAVYWGQRFDGLLRDTCKTNNYKIVFISFLDKFGCEIRKPELELEGVCGPSVGNPCSFLESQIKECQRMGVKVFLALGGPKGTYSACSADYAKDLAEYLHTYFLSERREGPLGKVYLDGLHFDIQK
PFDELIWDNLLEYLYQIKLVYQSTFLLSAAPGCLSFDEYLDNAIQTRHFDYIFVRIYNDRSCQYSTGNIQRIRNAWLIWTKSVYPRDWNLFLELPASQATAPGGGYIPPSALIYQVLVYLPDLQTRYAGIAL
WNRQADKETGYSTNIIRYLNATAMPFTSNLLKYPS

86. DISSTEIAVYWGQRLDGLLRDTCKTNNYKIVFISFLDKFGCEIRKPELELEGVCGPSVGNPCSFLESQIKECQRMGVKVFLALGGPKGTYSACSADYAKDLAEYLHTYFLSERREGPLGKVYLDGLHFDIQK
PFDELIWDNLLEYLYQIKFVYQSTFLLSAAPGCLSFDEYLDNAIQTRHFDYIFVRVYNDRSCQYSTGNIQRIRNAWLDWTKSVYPRDWNLFLELPASQATAPGGGYIPPSALIYQVLVYLPDLQTRYAGIA
LWNRQADKETGYSTNIIRYLNATAMPFTSNLLKYPS

87. DISSTEIAVYWGQRFDGLLRDTCKTNNYKIVFISFLDKFGCEIRKPELELEGVCGPSVGNPCSFLESQIKECQRMGVKVFLALGGPKGTYSACSADYAKDLAEYLHTYFLSERREGPLGKVYLDGLHFDIQK
PFDELIWDNLLEYLYQIKFVYQSTFLLSAAPGCLSFDEYLDNAIQTRHFDYIFVRVYNDRSCQYSTGNIQRIRNAWLDWTKSVYPRDWNLFLELPASQATAPGGGYIPPSALIYQVLVYLPDLQTRYAGIA
LWNRQADKETGYSTNIIRYLNATAMPFTSNLLKYPS

88. DISSTEIAVYWGQRLDGLLRDTCKTNNYKIVFISFLDKFGCEIRKPELELEGVCGPSVGNPCSFLESQIKECQRMGVKVFLALGGPKGTYSACSADYAKDLAEYLHTYFLSERREGPLGKVYLDGLHFDIQK
PFDELIWDNLLEYLYQIKLVYQSTFLLSAAPGCLSFDEYLDNAIQTRHFDYIFVRVYNDRSCQYSTGNIQRIRNAWLDWTKSVYPRDWNLFLELPASQATAPGGGYIPPSALIYQVLVYLPDLQTRYAGIA
LWNRQADKETGYSTNIIRYLNATAMPFTSNLLKYPS

89. DISSTEIAVYWGQRFDGLLRDTCKTNNYKIVFISFLDKFGCEIRKPELELEGVCGPSVGNPCSFLESQIKECQRMGVKVFLALGGPKGTYSACSADYAKDLAEYLHTYFLSERREGPLGKVYLDGLHFDIQK
PFDELIWDNLLEYLYQIKLVYQSTFLLSAAPGCLSFDEYLDNAIQTRHFDYIFVRVYNDRSCQYSTGNIQRIRNAWLDWTKSVYPRDWNLFLELPASQATAPGGGYIPPSALIYQVLVYLPDLQTRYAGIA
LWNRQADKETGYSTNIIRYLNATAMPFTSNLLKYPS

90. DISSTEIAVYWGQRLDGLLRDTCKTNNYKIVFISFLDKFGCEIRKPELELEGVCGPSVGNPCSFLESQIKECQRMGVKVFLALGGPKGTYSACSADYAKDLAEYLHTYFLSERREGPLGKVYLDGLHFDIQK
PFDELIWDNLLEYLYQIKFVYQSTFLLSAAPGCLSFDEYLDNAIQTRHFDYIFVRIYNDRSCQYSTGNIQRIRNAWLDWTKSVYPRDWNLFLELPASQATAPGGGYIPPSALIYQVLVYLPDLQTRYAGIAL
WNRQADKETGYSTNIIRYLNATAMPFTSNLLKYPS

91. DISSTEIAVYWGQRFDGLLRDTCKTNNYKIVFISFLDKFGCEIRKPELELEGVCGPSVGNPCSFLESQIKECQRMGVKVFLALGGPKGTYSACSADYAKDLAEYLHTYFLSERREGPLGKVYLDGLHFDIQK
PFDELIWDNLLEYLYQIKFVYQSTFLLSAAPGCLSFDEYLDNAIQTRHFDYIFVRIYNDRSCQYSTGNIQRIRNAWLDWTKSVYPRDWNLFLELPASQATAPGGGYIPPSALIYQVLVYLPDLQTRYAGIAL
WNRQADKETGYSTNIIRYLNATAMPFTSNLLKYPS

92. DISSTEIAVYWGQRLDGLLRDTCKTNNYKIVFISFLDKFGCEIRKPELELEGVCGPSVGNPCSFLESQIKECQRMGVKVFLALGGPKGTYSACSADYAKDLAEYLHTYFLSERREGPLGKVYLDGLHFDIQK
PFDELIWDNLLEYLYQIKLVYQSTFLLSAAPGCLSFDEYLDNAIQTRHFDYIFVRIYNDRSCQYSTGNIQRIRNAWLDWTKSVYPRDWNLFLELPASQATAPGGGYIPPSALIYQVLVYLPDLQTRYAGIAL
WNRQADKETGYSTNIIRYLNATAMPFTSNLLKYPS

93. DISSTEIAVYWGQRFDGLLRDTCKTNNYKIVFISFLDKFGCEIRKPELELEGVCGPSVGNPCSFLESQIKECQRMGVKVFLALGGPKGTYSACSADYAKDLAEYLHTYFLSERREGPLGKVYLDGLHFDIQK
PFDELIWDNLLEYLYQIKLVYQSTFLLSAAPGCLSFDEYLDNAIQTRHFDYIFVRIYNDRSCQYSTGNIQRIRNAWLDWTKSVYPRDWNLFLELPASQATAPGGGYIPPSALIYQVLVYLPDLQTRYAGIAL
WNRQADKETGYSTNIIRYLNATAMPFTSNLLKYPS

94. DISSTEIAVYWGQRLDGLLRDTCKTNNYKIVFISFLDKFGCEIRKPELELEGVCGPSVGNPCSFLESQIKECQRMGVKVFLALGGPKGTYSACSADYAKDLAEYLHTYFLSERREGPLGKVYLDGLHFDIQK
PFDELIWDNLLEYLYQIKFVYQSTFLLSAAPGCLSFDEYLDNAIQTRHFDYIFVRVYNDRSCQYSTGNIQRIRNAWLGWTKSVYPRDWNLFLELPASQATAPGGGYIPPSALIYQVLVYLPDLQTRYAGIA
LWNRQADKETGYSTNIIRYLNATAMPFTSNLLKYPS

95. DISSTEIAVYWGQRFDGLLRDTCKTNNYKIVFISFLDKFGCEIRKPELELEGVCGPSVGNPCSFLESQIKECQRMGVKVFLALGGPKGTYSACSADYAKDLAEYLHTYFLSERREGPLGKVYLDGLHFDIQK
PFDELIWDNLLEYLYQIKFVYQSTFLLSAAPGCLSFDEYLDNAIQTRHFDYIFVRVYNDRSCQYSTGNIQRIRNAWLGWTKSVYPRDWNLFLELPASQATAPGGGYIPPSALIYQVLVYLPDLQTRYAGIA
LWNRQADKETGYSTNIIRYLNATAMPFTSNLLKYPS

96. DISSTEIAVYWGQRLDGLLRDTCKTNNYKIVFISFLDKFGCEIRKPELELEGVCGPSVGNPCSFLESQIKECQRMGVKVFLALGGPKGTYSACSADYAKDLAEYLHTYFLSERREGPLGKVYLDGLHFDIQK
PFDELIWDNLLEYLYQIKLVYQSTFLLSAAPGCLSFDEYLDNAIQTRHFDYIFVRVYNDRSCQYSTGNIQRIRNAWLGWTKSVYPRDWNLFLELPASQATAPGGGYIPPSALIYQVLVYLPDLQTRYAGIA
LWNRQADKETGYSTNIIRYLNATAMPFTSNLLKYPS

97. DISSTEIAVYWGQRFDGLLRDTCKTNNYKIVFISFLDKFGCEIRKPELELEGVCGPSVGNPCSFLESQIKECQRMGVKVFLALGGPKGTYSACSADYAKDLAEYLHTYFLSERREGPLGKVYLDGLHFDIQK
PFDELIWDNLLEYLYQIKLVYQSTFLLSAAPGCLSFDEYLDNAIQTRHFDYIFVRVYNDRSCQYSTGNIQRIRNAWLGWTKSVYPRDWNLFLELPASQATAPGGGYIPPSALIYQVLVYLPDLQTRYAGIA
LWNRQADKETGYSTNIIRYLNATAMPFTSNLLKYPS

98. DISSTEIAVYWGQRLDGLLRDTCKTNNYKIVFISFLDKFGCEIRKPELELEGVCGPSVGNPCSFLESQIKECQRMGVKVFLALGGPKGTYSACSADYAKDLAEYLHTYFLSERREGPLGKVYLDGLHFDIQK
PFDELIWDNLLEYLYQIKFVYQSTFLLSAAPGCLSFDEYLDNAIQTRHFDYIFVRIYNDRSCQYSTGNIQRIRNAWLGWTKSVYPRDWNLFLELPASQATAPGGGYIPPSALIYQVLVYLPDLQTRYAGIAL
WNRQADKETGYSTNIIRYLNATAMPFTSNLLKYPS

99. DISSTEIAVYWGQRFDGLLRDTCKTNNYKIVFISFLDKFGCEIRKPELELEGVCGPSVGNPCSFLESQIKECQRMGVKVFLALGGPKGTYSACSADYAKDLAEYLHTYFLSERREGPLGKVYLDGLHFDIQK
PFDELIWDNLLEYLYQIKFVYQSTFLLSAAPGCLSFDEYLDNAIQTRHFDYIFVRIYNDRSCQYSTGNIQRIRNAWLGWTKSVYPRDWNLFLELPASQATAPGGGYIPPSALIYQVLVYLPDLQTRYAGIAL
WNRQADKETGYSTNIIRYLNATAMPFTSNLLKYPS

100. DISSTEIAVYWGQRLDGLLRDTCKTNNYKIVFISFLDKFGCEIRKPELELEGVCGPSVGNPCSFLESQIKECQRMGVKVFLALGGPKGTYSACSADYAKDLAEYLHTYFLSERREGPLGKVYLDGLHFDIQK
PFDELIWDNLLEYLYQIKLVYQSTFLLSAAPGCLSFDEYLDNAIQTRHFDYIFVRIYNDRSCQYSTGNIQRIRNAWLGWTKSVYPRDWNLFLELPASQATAPGGGYIPPSALIYQVLVYLPDLQTRYAGIAL
WNRQADKETGYSTNIIRYLNATAMPFTSNLLKYPS

101. DISSTEIAVYWGQRFDGLLRDTCKTNNYKIVFISFLDKFGCEIRKPELELEGVCGPSVGNPCSFLESQIKECQRMGVKVFLALGGPKGTYSACSADYAKDLAEYLHTYFLSERREGPLGKVYLDGLHFDIQK
PFDELIWDNLLEYLYQIKLVYQSTFLLSAAPGCLSFDEYLDNAIQTRHFDYIFVRIYNDRSCQYSTGNIQRIRNAWLGWTKSVYPRDWNLFLELPASQATAPGGGYIPPSALIYQVLVYLPDLQTRYAGIAL
WNRQADKETGYSTNIIRYLNATAMPFTSNLLKYPS

102. DISSTEIAVYWGQRLDGLLRDTCKTNNYKIVFISFLDKFGCEIRKPELELEGVCGPSVGNPCSFLESQIKECQRMGVKVFLALGGPKGTYSACSADYAKDLAEYLHTYFLSERREGPLGKVYLDGLHFDIQK
PFDELGWDNLLEYLYQIKFVYQSTFLLSAAPGCLSFDEYLDNAIQTRHFDYIFVRVYNDRSCQYSTGNIQRIRNAWLIWTKSVYPRDWNLFLELPASQATAPGGGYIPPSALIYQVLVYLPDLQTRYAGIA
LWNRQADKETGYSTNIIRYLNATAMPFTSNLLKYPS

103. DISSTEIAVYWGQRFDGLLRDTCKTNNYKIVFISFLDKFGCEIRKPELELEGVCGPSVGNPCSFLESQIKECQRMGVKVFLALGGPKGTYSACSADYAKDLAEYLHTYFLSERREGPLGKVYLDGLHFDIQK
PFDELGWDNLLEYLYQIKFVYQSTFLLSAAPGCLSFDEYLDNAIQTRHFDYIFVRVYNDRSCQYSTGNIQRIRNAWLIWTKSVYPRDWNLFLELPASQATAPGGGYIPPSALIYQVLVYLPDLQTRYAGIA
LWNRQADKETGYSTNIIRYLNATAMPFTSNLLKYPS

104. DISSTEIAVYWGQRLDGLLRDTCKTNNYKIVFISFLDKFGCEIRKPELELEGVCGPSVGNPCSFLESQIKECQRMGVKVFLALGGPKGTYSACSADYAKDLAEYLHTYFLSERREGPLGKVYLDGLHFDIQK
PFDELGWDNLLEYLYQIKLVYQSTFLLSAAPGCLSFDEYLDNAIQTRHFDYIFVRVYNDRSCQYSTGNIQRIRNAWLIWTKSVYPRDWNLFLELPASQATAPGGGYIPPSALIYQVLVYLPDLQTRYAGIA
LWNRQADKETGYSTNIIRYLNATAMPFTSNLLKYPS

105. DISSTEIAVYWGQRFDGLLRDTCKTNNYKIVFISFLDKFGCEIRKPELELEGVCGPSVGNPCSFLESQIKECQRMGVKVFLALGGPKGTYSACSADYAKDLAEYLHTYFLSERREGPLGKVYLDGLHFDIQK
PFDELGWDNLLEYLYQIKLVYQSTFLLSAAPGCLSFDEYLDNAIQTRHFDYIFVRVYNDRSCQYSTGNIQRIRNAWLIWTKSVYPRDWNLFLELPASQATAPGGGYIPPSALIYQVLVYLPDLQTRYAGIA
LWNRQADKETGYSTNIIRYLNATAMPFTSNLLKYPS

106. DISSTEIAVYWGQRLDGLLRDTCKTNNYKIVFISFLDKFGCEIRKPELELEGVCGPSVGNPCSFLESQIKECQRMGVKVFLALGGPKGTYSACSADYAKDLAEYLHTYFLSERREGPLGKVYLDGLHFDIQK
PFDELGWDNLLEYLYQIKFVYQSTFLLSAAPGCLSFDEYLDNAIQTRHFDYIFVRIYNDRSCQYSTGNIQRIRNAWLIWTKSVYPRDWNLFLELPASQATAPGGGYIPPSALIYQVLVYLPDLQTRYAGIAL
WNRQADKETGYSTNIIRYLNATAMPFTSNLLKYPS

107. DISSTEIAVYWGQRFDGLLRDTCKTNNYKIVFISFLDKFGCEIRKPELELEGVCGPSVGNPCSFLESQIKECQRMGVKVFLALGGPKGTYSACSADYAKDLAEYLHTYFLSERREGPLGKVYLDGLHFDIQK
PFDELGWDNLLEYLYQIKFVYQSTFLLSAAPGCLSFDEYLDNAIQTRHFDYIFVRIYNDRSCQYSTGNIQRIRNAWLIWTKSVYPRDWNLFLELPASQATAPGGGYIPPSALIYQVLVYLPDLQTRYAGIAL
WNRQADKETGYSTNIIRYLNATAMPFTSNLLKYPS

108. DISSTEIAVYWGQRLDGLLRDTCKTNNYKIVFISFLDKFGCEIRKPELELEGVCGPSVGNPCSFLESQIKECQRMGVKVFLALGGPKGTYSACSADYAKDLAEYLHTYFLSERREGPLGKVYLDGLHFDIQK
PFDELGWDNLLEYLYQIKLVYQSTFLLSAAPGCLSFDEYLDNAIQTRHFDYIFVRIYNDRSCQYSTGNIQRIRNAWLIWTKSVYPRDWNLFLELPASQATAPGGGYIPPSALIYQVLVYLPDLQTRYAGIAL
WNRQADKETGYSTNIIRYLNATAMPFTSNLLKYPS

109. DISSTEIAVYWGQRFDGLLRDTCKTNNYKIVFISFLDKFGCEIRKPELELEGVCGPSVGNPCSFLESQIKECQRMGVKVFLALGGPKGTYSACSADYAKDLAEYLHTYFLSERREGPLGKVYLDGLHFDIQK
PFDELGWDNLLEYLYQIKLVYQSTFLLSAAPGCLSFDEYLDNAIQTRHFDYIFVRIYNDRSCQYSTGNIQRIRNAWLIWTKSVYPRDWNLFLELPASQATAPGGGYIPPSALIYQVLVYLPDLQTRYAGIAL
WNRQADKETGYSTNIIRYLNATAMPFTSNLLKYPS

110. DISSTEIAVYWGQRLDGLLRDTCKTNNYKIVFISFLDKFGCEIRKPELELEGVCGPSVGNPCSFLESQIKECQRMGVKVFLALGGPKGTYSACSADYAKDLAEYLHTYFLSERREGPLGKVYLDGLHFDIQK
PFDELGWDNLLEYLYQIKFVYQSTFLLSAAPGCLSFDEYLDNAIQTRHFDYIFVRVYNDRSCQYSTGNIQRIRNAWLDWTKSVYPRDWNLFLELPASQATAPGGGYIPPSALIYQVLVYLPDLQTRYAGIA
LWNRQADKETGYSTNIIRYLNATAMPFTSNLLKYPS

111. DISSTEIAVYWGQRFDGLLRDTCKTNNYKIVFISFLDKFGCEIRKPELELEGVCGPSVGNPCSFLESQIKECQRMGVKVFLALGGPKGTYSACSADYAKDLAEYLHTYFLSERREGPLGKVYLDGLHFDIQK
PFDELGWDNLLEYLYQIKFVYQSTFLLSAAPGCLSFDEYLDNAIQTRHFDYIFVRVYNDRSCQYSTGNIQRIRNAWLDWTKSVYPRDWNLFLELPASQATAPGGGYIPPSALIYQVLVYLPDLQTRYAGIA
LWNRQADKETGYSTNIIRYLNATAMPFTSNLLKYPS

112. DISSTEIAVYWGQRLDGLLRDTCKTNNYKIVFISFLDKFGCEIRKPELELEGVCGPSVGNPCSFLESQIKECQRMGVKVFLALGGPKGTYSACSADYAKDLAEYLHTYFLSERREGPLGKVYLDGLHFDIQK
PFDELGWDNLLEYLYQIKLVYQSTFLLSAAPGCLSFDEYLDNAIQTRHFDYIFVRVYNDRSCQYSTGNIQRIRNAWLDWTKSVYPRDWNLFLELPASQATAPGGGYIPPSALIYQVLVYLPDLQTRYAGIA
LWNRQADKETGYSTNIIRYLNATAMPFTSNLLKYPS

113. DISSTEIAVYWGQRFDGLLRDTCKTNNYKIVFISFLDKFGCEIRKPELELEGVCGPSVGNPCSFLESQIKECQRMGVKVFLALGGPKGTYSACSADYAKDLAEYLHTYFLSERREGPLGKVYLDGLHFDIQK
PFDELGWDNLLEYLYQIKLVYQSTFLLSAAPGCLSFDEYLDNAIQTRHFDYIFVRVYNDRSCQYSTGNIQRIRNAWLDWTKSVYPRDWNLFLELPASQATAPGGGYIPPSALIYQVLVYLPDLQTRYAGIA
LWNRQADKETGYSTNIIRYLNATAMPFTSNLLKYPS

114. DISSTEIAVYWGQRLDGLLRDTCKTNNYKIVFISFLDKFGCEIRKPELELEGVCGPSVGNPCSFLESQIKECQRMGVKVFLALGGPKGTYSACSADYAKDLAEYLHTYFLSERREGPLGKVYLDGLHFDIQK
PFDELGWDNLLEYLYQIKFVYQSTFLLSAAPGCLSFDEYLDNAIQTRHFDYIFVRIYNDRSCQYSTGNIQRIRNAWLDWTKSVYPRDWNLFLELPASQATAPGGGYIPPSALIYQVLVYLPDLQTRYAGIA
LWNRQADKETGYSTNIIRYLNATAMPFTSNLLKYPS

115. DISSTEIAVYWGQRFDGLLRDTCKTNNYKIVFISFLDKFGCEIRKPELELEGVCGPSVGNPCSFLESQIKECQRMGVKVFLALGGPKGTYSACSADYAKDLAEYLHTYFLSERREGPLGKVYLDGLHFDIQK
PFDELGWDNLLEYLYQIKFVYQSTFLLSAAPGCLSFDEYLDNAIQTRHFDYIFVRIYNDRSCQYSTGNIQRIRNAWLDWTKSVYPRDWNLFLELPASQATAPGGGYIPPSALIYQVLVYLPDLQTRYAGIA
LWNRQADKETGYSTNIIRYLNATAMPFTSNLLKYPS

116. DISSTEIAVYWGQRLDGLLRDTCKTNNYKIVFISFLDKFGCEIRKPELELEGVCGPSVGNPCSFLESQIKECQRMGVKVFLALGGPKGTYSACSADYAKDLAEYLHTYFLSERREGPLGKVYLDGLHFDIQK
PFDELGWDNLLEYLYQIKLVYQSTFLLSAAPGCLSFDEYLDNAIQTRHFDYIFVRIYNDRSCQYSTGNIQRIRNAWLDWTKSVYPRDWNLFLELPASQATAPGGGYIPPSALIYQVLVYLPDLQTRYAGIA
LWNRQADKETGYSTNIIRYLNATAMPFTSNLLKYPS

117. DISSTEIAVYWGQRFDGLLRDTCKTNNYKIVFISFLDKFGCEIRKPELELEGVCGPSVGNPCSFLESQIKECQRMGVKVFLALGGPKGTYSACSADYAKDLAEYLHTYFLSERREGPLGKVYLDGLHFDIQK
PFDELGWDNLLEYLYQIKLVYQSTFLLSAAPGCLSFDEYLDNAIQTRHFDYIFVRIYNDRSCQYSTGNIQRIRNAWLDWTKSVYPRDWNLFLELPASQATAPGGGYIPPSALIYQVLVYLPDLQTRYAGIA
LWNRQADKETGYSTNIIRYLNATAMPFTSNLLKYPS

118. DISSTEIAVYWGQRLDGLLRDTCKTNNYKIVFISFLDKFGCEIRKPELELEGVCGPSVGNPCSFLESQIKECQRMGVKVFLALGGPKGTYSACSADYAKDLAEYLHTYFLSERREGPLGKVYLDGLHFDIQK
PFDELGWDNLLEYLYQIKFVYQSTFLLSAAPGCLSFDEYLDNAIQTRHFDYIFVRVYNDRSCQYSTGNIQRIRNAWLGWTKSVYPRDWNLFLELPASQATAPGGGYIPPSALIYQVLVYLPDLQTRYAGIA
LWNRQADKETGYSTNIIRYLNATAMPFTSNLLKYPS

119. DISSTEIAVYWGQRFDGLLRDTCKTNNYKIVFISFLDKFGCEIRKPELELEGVCGPSVGNPCSFLESQIKECQRMGVKVFLALGGPKGTYSACSADYAKDLAEYLHTYFLSERREGPLGKVYLDGLHFDIQK
PFDELGWDNLLEYLYQIKFVYQSTFLLSAAPGCLSFDEYLDNAIQTRHFDYIFVRVYNDRSCQYSTGNIQRIRNAWLGWTKSVYPRDWNLFLELPASQATAPGGGYIPPSALIYQVLVYLPDLQTRYAGIA
LWNRQADKETGYSTNIIRYLNATAMPFTSNLLKYPS

120. DISSTEIAVYWGQRLDGLLRDTCKTNNYKIVFISFLDKFGCEIRKPELELEGVCGPSVGNPCSFLESQIKECQRMGVKVFLALGGPKGTYSACSADYAKDLAEYLHTYFLSERREGPLGKVYLDGLHFDIQK
PFDELGWDNLLEYLYQIKLVYQSTFLLSAAPGCLSFDEYLDNAIQTRHFDYIFVRVYNDRSCQYSTGNIQRIRNAWLGWTKSVYPRDWNLFLELPASQATAPGGGYIPPSALIYQVLVYLPDLQTRYAGIA
LWNRQADKETGYSTNIIRYLNATAMPFTSNLLKYPS

121. DISSTEIAVYWGQRFDGLLRDTCKTNNYKIVFISFLDKFGCEIRKPELELEGVCGPSVGNPCSFLESQIKECQRMGVKVFLALGGPKGTYSACSADYAKDLAEYLHTYFLSERREGPLGKVYLDGLHFDIQK
PFDELGWDNLLEYLYQIKLVYQSTFLLSAAPGCLSFDEYLDNAIQTRHFDYIFVRVYNDRSCQYSTGNIQRIRNAWLGWTKSVYPRDWNLFLELPASQATAPGGGYIPPSALIYQVLVYLPDLQTRYAGIA
LWNRQADKETGYSTNIIRYLNATAMPFTSNLLKYPS

122. DISSTEIAVYWGQRLDGLLRDTCKTNNYKIVFISFLDKFGCEIRKPELELEGVCGPSVGNPCSFLESQIKECQRMGVKVFLALGGPKGTYSACSADYAKDLAEYLHTYFLSERREGPLGKVYLDGLHFDIQK
PFDELGWDNLLEYLYQIKFVYQSTFLLSAAPGCLSFDEYLDNAIQTRHFDYIFVRIYNDRSCQYSTGNIQRIRNAWLGWTKSVYPRDWNLFLELPASQATAPGGGYIPPSALIYQVLVYLPDLQTRYAGIA
LWNRQADKETGYSTNIIRYLNATAMPFTSNLLKYPS

123. DISSTEIAVYWGQRFDGLLRDTCKTNNYKIVFISFLDKFGCEIRKPELELEGVCGPSVGNPCSFLESQIKECQRMGVKVFLALGGPKGTYSACSADYAKDLAEYLHTYFLSERREGPLGKVYLDGLHFDIQK
PFDELGWDNLLEYLYQIKFVYQSTFLLSAAPGCLSFDEYLDNAIQTRHFDYIFVRIYNDRSCQYSTGNIQRIRNAWLGWTKSVYPRDWNLFLELPASQATAPGGGYIPPSALIYQVLVYLPDLQTRYAGIA
LWNRQADKETGYSTNIIRYLNATAMPFTSNLLKYPS

124. DISSTEIAVYWGQRLDGLLRDTCKTNNYKIVFISFLDKFGCEIRKPELELEGVCGPSVGNPCSFLESQIKECQRMGVKVFLALGGPKGTYSACSADYAKDLAEYLHTYFLSERREGPLGKVYLDGLHFDIQK
PFDELGWDNLLEYLYQIKLVYQSTFLLSAAPGCLSFDEYLDNAIQTRHFDYIFVRIYNDRSCQYSTGNIQRIRNAWLGWTKSVYPRDWNLFLELPASQATAPGGGYIPPSALIYQVLVYLPDLQTRYAGIA
LWNRQADKETGYSTNIIRYLNATAMPFTSNLLKYPS

125. DISSTEIAVYWGQRFDGLLRDTCKTNNYKIVFISFLDKFGCEIRKPELELEGVCGPSVGNPCSFLESQIKECQRMGVKVFLALGGPKGTYSACSADYAKDLAEYLHTYFLSERREGPLGKVYLDGLHFDIQK
PFDELGWDNLLEYLYQIKLVYQSTFLLSAAPGCLSFDEYLDNAIQTRHFDYIFVRIYNDRSCQYSTGNIQRIRNAWLGWTKSVYPRDWNLFLELPASQATAPGGGYIPPSALIYQVLVYLPDLQTRYAGIA
LWNRQADKETGYSTNIIRYLNATAMPFTSNLLKYPS

The sequences showed in blue color are the 5 modified versions closer to native Concanavalin B (Stability, free folding energy and total contacts.

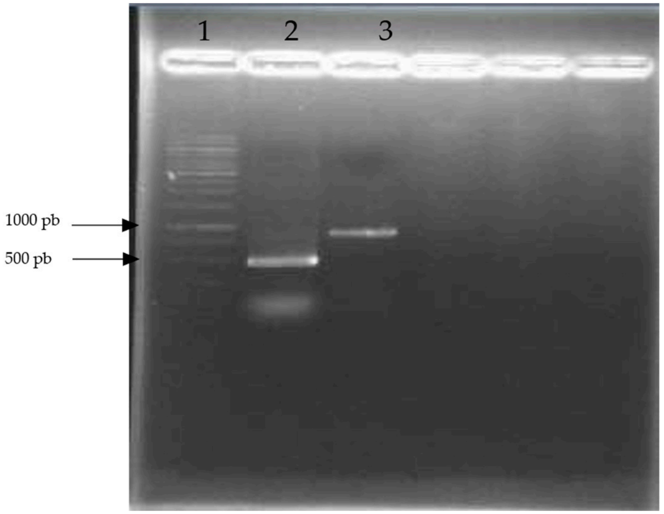

Figure S1. Agarose gel of the PCR product of concanavalin B extracted from leaves. Lane 1: Molecular marker, Lane 2: Positive control, Lane 3: product of PCR.

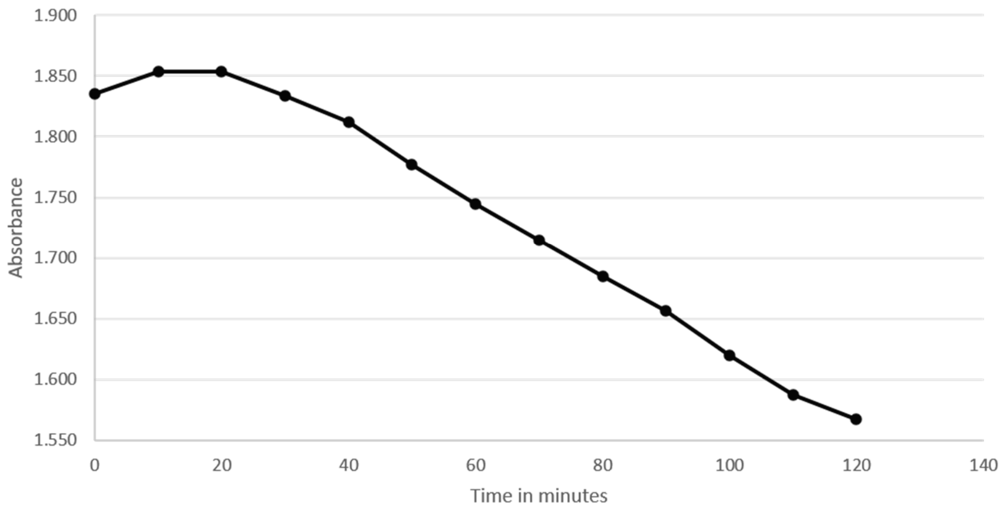

Figure S2. NADPH stability in phosphate buffer pH 7.4 and EDTA 200 mM.
